# Major β cell-specific functions of NKX2.2 are mediated via the NK2-specific domain

**DOI:** 10.1101/gad.350569.123

**Published:** 2023-06-01

**Authors:** Vladimir Abarinov, Joshua A. Levine, Angela J. Churchill, Bryce Hopwood, Cailin S. Deiter, Michelle A. Guney, Kristen L. Wells, Jessica M. Schrunk, Yuchun Guo, Jennifer Hammelman, David K. Gifford, Mark A. Magnuson, Hynek Wichterle, Lori Sussel

**Affiliations:** 1 Department of Genetics and Development, Columbia University, New York, New York 10032, USA;; 2 Barbara Davis Center for Diabetes, University of Colorado Anschutz Medical Campus, Aurora, Colorado 80045, USA;; 3 Computer Science and Artificial Intelligence Laboratory, Massachusetts Institute of Technology, Cambridge, Massachusetts 02139, USA;; 4 Department of Molecular Physiology and Biophysics, Center for Stem Cell Biology, Vanderbilt University School of Medicine, Nashville, Tennessee 37232, USA;; 5 Department of Pathology and Cell Biology, Columbia University, New York, New York 10032, USA;; 6 Department of Neurology, Columbia University, New York, New York 10032, USA;; 7 Department of Neuroscience, Columbia University, New York, New York 10032, USA

**Keywords:** β cells, NKX2.2, pancreatic islet, spinal cord, transcriptional regulation

## Abstract

The consolidation of unambiguous cell fate commitment relies on the ability of transcription factors (TFs) to exert tissue-specific regulation of complex genetic networks. However, the mechanisms by which TFs establish such precise control over gene expression have remained elusive—especially in instances in which a single TF operates in two or more discrete cellular systems. In this study, we demonstrate that β cell-specific functions of NKX2.2 are driven by the highly conserved NK2-specific domain (SD). Mutation of the endogenous NKX2.2 SD prevents the developmental progression of β cell precursors into mature, insulin-expressing β cells, resulting in overt neonatal diabetes. Within the adult β cell, the SD stimulates β cell performance through the activation and repression of a subset of NKX2.2-regulated transcripts critical for β cell function. These irregularities in β cell gene expression may be mediated via SD-contingent interactions with components of chromatin remodelers and the nuclear pore complex. However, in stark contrast to these pancreatic phenotypes, the SD is entirely dispensable for the development of NKX2.2-dependent cell types within the CNS. Together, these results reveal a previously undetermined mechanism through which NKX2.2 directs disparate transcriptional programs in the pancreas versus neuroepithelium.

## Abstract (precis)

In this study, Abarinov et al. describe how the transcription factor NKX2.2 affects tissue-specific transcriptional programs. They show that NKX2.2's NK2-specific domain is essential for its role in pancreatic β cell, but not neuroepithelial, development and function, providing insight into the etiology of early-onset diabetes.

Differentiation of multicellular organisms relies on the implementation and execution of unique, tissue-specific transcriptional programs established during development. This process is dependent on the ability of transcription factors (TFs) to achieve precise regulation of cell-specific target genes. Although the general influence of a TF's DNA binding motif, its underlying chromatin structure, and the presence of tissue-specific cofactors can contribute to DNA binding selectivity (Mann et al. 2009; Calo and Wysocka 2013; Meredith et al. 2013; Whyte et al. 2013; Iwafuchi-Doi and Zaret 2014; Long et al. 2016; Bobola and Merabet 2017), these elements often cannot fully account for a TF's functional specificity. Indeed, achieving selective regulation becomes further complicated in cell types in which a single TF acts as both a transcriptional activator and repressor and/or controls the genetic networks of two or more highly divergent tissue systems.

To address how TFs play distinct roles in different cellular contexts, we examined the pancreas and central nervous system (CNS)—two tissues that share several common TFs, each of which is essential for unique cell specification decisions within the two systems (Ericson et al. 1997; Sussel et al. 1998; Briscoe et al. 1999, 2000; Jessell 2000; Sander et al. 2000a,b; Jørgensen et al. 2007; Whyte et al. 2013). Specifically, we focused on the strategies by which NKX2.2 drives cell fate determination, as deletion of NKX2.2 in either the pancreas or CNS results in tissue-intrinsic cell fate conversions (Briscoe et al. 1999; Prado et al. 2004). In the developing pancreas, NKX2.2 is expressed in pancreatic progenitors that give rise to six different endocrine cell populations (Sussel et al. 1998; Prado et al. 2004; Arnes et al. 2012; Suissa et al. 2013). In *Nkx2.2*-null embryos, insulin-producing β cells fail to form entirely, and there is a significant reduction in glucagon-expressing α cells (Sussel et al. 1998; Prado et al. 2004). The loss of these two cell types is accompanied by a concomitant increase in the ghrelin-expressing ε cell lineage (Prado et al. 2004). Analogously, in the developing spinal cord, NKX2.2 is expressed in p3 progenitors, which give rise to V3 interneurons (INs). In the absence of NKX2.2, V3 populations fail to form, and a concurrent expansion of motor neuron (MN) generation is observed (Briscoe et al. 1999).

In addition to its homeodomain, NKX2.2 contains two other highly conserved regions: the tinman (TN) domain and the NK2-specific domain (SD) (Smith and Jaynes 1996; Watada et al. 2000; Koizumi et al. 2003). Extensive loss- and gain-of-function studies have implicated the TN domain in the execution of both NKX2.2 pancreatic and neural activities (Muhr et al. 2001; Papizan et al. 2011). This domain facilitates the interaction between NKX2.2 and the Groucho (GRG) family of corepressor proteins (Muhr et al. 2001; Doyle et al. 2007; Papizan et al. 2011). When the TN domain is mutated, the interaction between NKX2.2 and GRG is disrupted, and NKX2.2 can no longer properly specify endocrine or neuronal lineages (Muhr et al. 2001; Papizan et al. 2011). Together, these studies highlight the importance of the GRG corepressors in mediating the activity of NKX2.2 in both organ systems. However, how NKX2.2 achieves selective regulation of pancreatic versus neural genetic programs remains unknown. Unlike the TN domain, the function of the SD has not been well characterized. The SD represents the defining feature of the NK2 family of TFs and is 95% conserved among species (Fig. 1A; Watada et al. 2000). Studies describing the function of this domain have been predominantly limited to in vitro model systems and have yielded conflicting results (Watada et al. 2000; Stepchenko and Nirenberg 2004; Uhler et al. 2007). To investigate the role of the SD more definitively, we generated mice in which conserved amino acids within the SD were replaced with alanine residues at the endogenous *Nkx2.2* locus and assessed the effect of this mutation on NKX2.2-dependent pancreatic and CNS populations.

**Figure 1.**
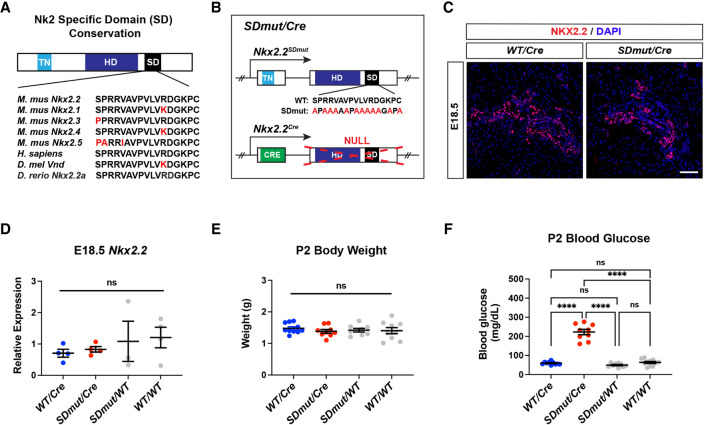
*Nkx2.2^SDmut/Cre^* animals develop overt neonatal diabetes. (*A*) Schematic highlighting amino acid conservation of the NK2-specific domain (SD). (*B*) Diagram of *Nkx2.2* alleles used to generate *Nkx2.2^SDmut/Cre^* mice. (*C*) NKX2.2 protein expression is similar in *Nkx2.2^Cre/+^* (WT/*Cre*) and *Nkx2.2^SDmut/Cre^* (*SDmut/Cre*) pancreata at E18.5. (*D*) E18.5 *Nkx2.2* mRNA expression remains unchanged among wild-type, heterozygous, and *Nkx2.2^SDmut/Cre^* pancreata. (*E*,*F*) Body weight (*E*) and blood glucose levels (*F*) of P2 neonates. Data are presented as mean ± SEM. (ns) Not significant, (****) *P* < 0.0001. Scale bar, 50 µm.

SD mutant mice showed striking endocrine defects: Mutant embryos exhibited a significant decrease in β cell formation arising from arrested β cell precursor differentiation and died shortly after birth due to severe neonatal diabetes. Furthermore, mutation of the SD specifically within β cells resulted in impaired function and rapid onset of hyperglycemia. In contrast, extensive phenotypic characterization in the CNS revealed few, if any, differences between SD mutant and control animals. These findings highlight a previously uncharacterized role for the NKX2.2 SD in conferring β cell-specific functions to the NKX2.2 protein and begin to elucidate how regions outside of TF DNA-binding domains establish tissue-specific gene regulatory networks.

## Results

### Generation of *Nkx2.2^SDmut^* mice

To determine the function of the NK2-specific domain (SD), we generated mice harboring a mutant allele (*Nkx2.2^SDmut^*) containing alanine substitutions within the SD region (Fig. 1B; Supplemental Fig. S1A). Given the high degree of conservation, the majority of the SD was replaced, leaving only structural amino acids intact (Fig. 1A,B). Similar to previously published *Nkx2.2* mutations, the *Nkx2.2^SDmut^* allele did not exhibit haploinsufficiency (Fig. 1C–F; Sussel et al. 1998; Papizan et al. 2011; Arnes et al. 2012; Balderes et al. 2013). We also introduced the *Nkx2.2^Cre^* allele into these mice to enable lineage tracing when examining phenotypes in the CNS (Fig. 1B). As the *Nkx2.2^Cre^* allele represents a null mutation (Balderes et al. 2013), *Nkx2.2^SDmut/Cre^* (*SDmut/Cre*) and *Nkx2.2^Cre/+^* (WT/*Cre*) genotypes denote *Nkx2.2^SDmut/−^* and *Nkx2.2^+/−^* animals, respectively (Fig. 1B).

Analogous to *Nkx2.2*-null animals, *Nkx2.2^SDmut/Cre^* mice were born at normal Mendelian ratios with no difference in body weight (Fig. 1E; Supplemental Fig. S1B). By P2, however, mutants exhibited overt hyperglycemia that worsened at P3 and died shortly thereafter (Fig. 1F; Supplemental Fig. S1C,D). Importantly, *Nkx2.2* mRNA levels and expression patterns in *Nkx2.2^SDmut/Cre^* embryos were similar to those of *Nkx2.2^Cre/+^* animals at E18.5 (Fig. 1C,D), suggesting that the neonatal hyperglycemia in *Nkx2.2^SDmut/Cre^* mice did not arise from a loss of NKX2.2 expression during development.

### *Nkx2.2^SDmut/Cre^* mice display altered endocrine specification

**Figure 2.**
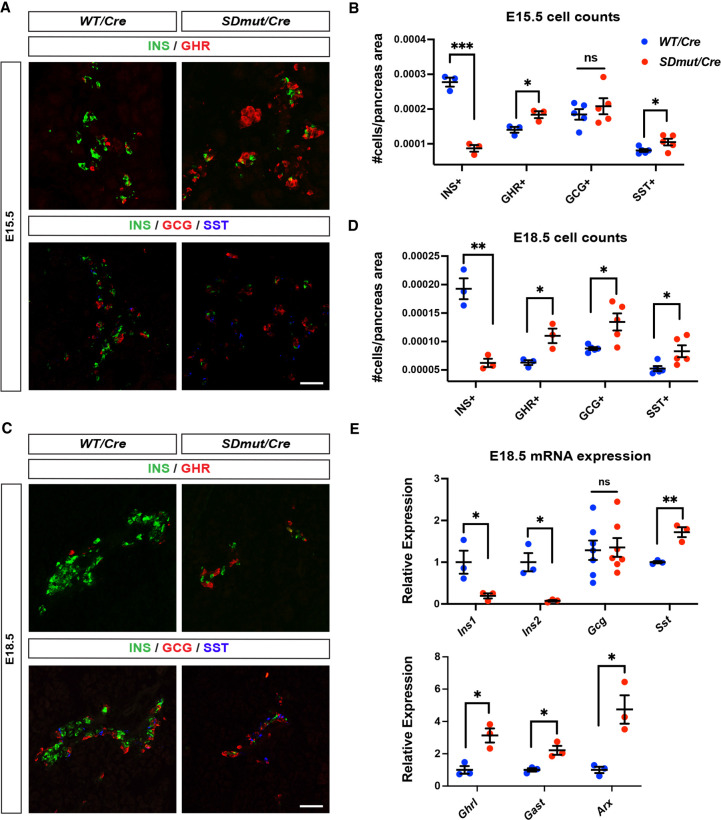
*Nkx2.2^SDmut/Cre^* embryos show an expansion of other endocrine cell types at the expense of β cells beginning at E15.5. (*A*–*E*) Characterization of insulin (INS^+^), glucagon (GCG^+^), ghrelin (GHR^+^), and somatostatin (SST^+^) cells in *Nkx2.2^SDmut/Cre^* (*SDmut/Cre*) and *Nkx2.2^Cre/+^* (WT/*Cre*) embryos. (*A*,*B*) Immunostaining (*A*) and quantification (*B*) of endocrine cell types in E15.5 embryos. (*C*–*E*) Immunostaining (*C*), quantification (*D*), and qRT-PCR analysis (*E*) at E18.8. Data are presented as mean ± SEM. (ns) Not significant, (*) *P* < 0.05, (**) *P* < 0.01, (***) *P* < 0.001. Scale bars represent 50 µm.

NKX2.2 is expressed in pancreatic progenitors and is required to regulate the specification of several endocrine cell lineages (Sussel et al. 1998; Prado et al. 2004; Churchill et al. 2017). In the absence of NKX2.2, embryos show a complete lack of β cells and a large decrease in α cells at the onset of endocrine differentiation (Sussel et al. 1998; Prado et al. 2004). The loss of these two populations is accompanied by a compensatory expansion of ghrelin-producing cells that is evident by E12.5 (Prado et al. 2004). To determine whether the severe neonatal diabetes associated with the SD mutation is caused by similar disruptions in cell fate specification, we assessed endocrine differentiation in *Nkx2.2^SDmut/Cre^* animals. Unlike *Nkx2.2^−/−^* mice, *Nkx2.2^SDmut/Cre^* embryos did not exhibit abnormalities in glucagon (GCG), ghrelin (GHR), or PDX1 expression at E12.5, indicating that the SD is not required to mediate NKX2.2 function during the earliest stages of β cell neogenesis (Supplemental Fig. S2A). During the peak of β cell differentiation at E15.5, however, *Nkx2.2^SDmut/Cre^* animals exhibited an ∼70% reduction in insulin-expressing (INS^+^) cells (Fig. 2A,B). As NEUROG3 expression and transcript levels appeared normal at this stage, the loss of β cells in *Nkx2.2^SDmut/Cre^* animals was likely not due to anomalies in endocrine progenitor generation (Supplemental Fig. S2B,C; Gradwohl et al. 2000). Interestingly, decreased β cell production occurred concurrently with an increase in not only GHR^+^ but also SST^+^ populations (Fig. 2A,B); SST^+^ cells have not previously been reported to be affected by *Nkx2.2* mutation during development (Sussel et al. 1998; Papizan et al. 2011; Arnes et al. 2012; Balderes et al. 2013; Churchill et al. 2017). These alterations to endocrine specification persisted through E18.5, at which time an increase in GCG^+^ cells was also observed (Fig. 2C,D). qRT-PCR analysis at E18.5 further confirmed a significant decrease in *Ins1/2* transcript levels concomitant with elevated *Sst*, *Ghrl*, *Gast*, and *Arx* gene expression (Fig. 2E). The reduction in INS^+^ cells and overall abnormalities in islet formation continued postnatally until death at approximately P2 (Supplemental Fig. S3A,B). Together, these data indicate that NKX2.2 uses the SD to repress alternative endocrine cell fates within the β cell lineage during the secondary phase of β cell neogenesis.

### β Cell precursors stall during differentiation in *Nkx2.2^SDmut/Cre^* mice

**Figure 3.**
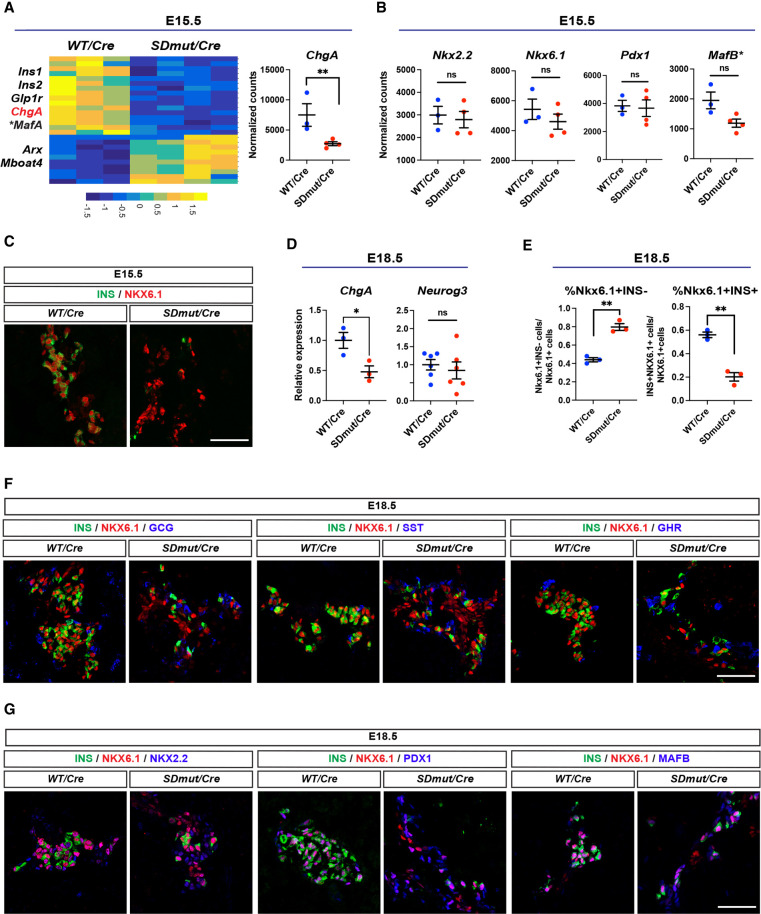
β Cells stall during differentiation in *Nkx2.2^SDmut/Cre^* embryos. (*A*, *left*) Heat map of significantly altered genes from RNA-seq analysis of E15.5 *Nkx2.2^Cre/+^* (WT/*Cre*) and *Nkx2.2^SDmut/Cre^* (*SDmut/Cre*) pancreata. (*Right*) Normalized expression of *ChgA*, a pan-endocrine marker. (*) *MafA* adjusted *P* value = 0.057. (*B*) Normalized expression of key transcription factors (TFs) needed for β cell differentiation. (*) *MafB* adjusted *P* value = 0.79 or nonadjusted *P* value = 0.0040. (*C*) Immunostaining confirmed NKX6.1 expression in the E15.5 mutant pancreas. (*D*) qRT-PCR of *ChgA* and *Neurog3* in E18.5 pancreata. (*E*) Percentage of NKX6.1^+^INS^−^ and NKX6.1^+^INS^+^ cells at E18.5. (*F*,*G*) NKX6.1^+^INS^−^ cells in *Nkx2.2^SDmut/Cre^* animals do not express GCG, SST, or GHR at E18.5 (*F*) but do express the β cell TFs NKX2.2, PDX1, and MAFB (*G*). Data are presented as mean ± SEM. (ns) Not significant, (*) *P* < 0.05, (**) *P* < 0.01. Scale bars, 50 µm.

To elucidate potential mechanisms underlying the disruption of endocrine specification caused by mutation of the SD, we performed RNA-seq on *Nkx2.2^Cre/+^* and *Nkx2.2^SDmut/Cre^* pancreata at E15.5. Confirming our phenotypic characterization, several β cell genes, including *Ins1/2* and *Glp1r*, were significantly reduced in mutant embryos, while ε cell-related transcripts were simultaneously up-regulated (Fig. 3A; Kordowich et al. 2011; Lim et al. 2011; Smith et al. 2014). However, transcriptional profiling also revealed that *ChgA*—a pan-endocrine gene found in all hormone-producing cells—was significantly decreased (Fig. 3A), suggesting that unlike all other previously examined *Nkx2.2* mutants (Sussel et al. 1998; Prado et al. 2004; Papizan et al. 2011; Churchill et al. 2017; Gutiérrez et al. 2017), the loss of β cell production in *Nkx2.2^SDmut/Cre^* animals was not entirely compensated for by a commensurate expansion of other endocrine populations. Interestingly, despite the reduction of *Ins1/2* at E15.5, *Nkx2.2^SDmut/Cre^* embryos exhibited no changes in mRNA levels of several TFs required for β cell development, including *Nkx2.2*, *Nkx6.1*, *MafB*, and *Pdx1* (Fig. 3B; Jonsson et al. 1994; Sussel et al. 1998; Sander et al. 2000b; Artner et al. 2007; Gannon et al. 2008; Gao et al. 2014). As INS production is one of the relatively later steps in β cell differentiation, these results suggest that the initial specification of β cell identity occurs in *Nkx2.2^SDmut/Cre^* mice. Consistent with this finding, immunostaining revealed that while the overall generation of NKX6.1^+^ cells appeared normal in *Nkx2.2^SDmut/Cre^* animals at E15.5, the majority of these cells lacked INS expression (Fig. 3C).

During endocrine specification, NKX6.1 is initially expressed in NEUROG3^+^ progenitors. After this stage of development, however, NKX6.1 becomes expressed exclusively within β cells (Sander et al. 2000b). To assess the extent of β cell differentiation in *Nkx2.2^SDmut/Cre^* embryos, we further characterized NKX6.1 expression at E18.5, when endocrine progenitors have largely disappeared and NKX6.1 expression is confined to the β cell lineage (Apelqvist et al. 1999; Gradwohl et al. 2000). Importantly, *Neurog3* transcript levels were not significantly different between *Nkx2.2^Cre/+^* and *Nkx2.2^SDmut/Cre^* pancreata at E18.5, indicating that mutation of the SD did not result in abnormal maintenance of NEUROG3^+^ progenitors (Fig. 3D).

Similar to our results at E15.5, while NKX6.1 expression appeared normal in *Nkx2.2^SDmut/Cre^* embryos at E18.5, the majority of NKX6.1*^+^* cells were deficient in INS expression (Fig. 3E,F). NKX6.1^+^INS^−^ cells were also GCG^−^, GHR^−^, and SST^−^ (Fig. 3F) and expressed the TFs PDX1, MAFB, and NKX2.2 (Fig. 3G). The combinatorial expression of these TFs is unique to embryonic β cells and serves as a strong indication of early β cell specification (Sussel et al. 1998; Artner et al. 2006; Nishimura et al. 2006; Babu et al. 2007; Arnes et al. 2012). These data suggest that the NKX6.1^+^INS^−^ cells in *Nkx2.2^SDmut/Cre^* embryos delineate a population of β cell precursors that have exited the NEUROG3^+^ endocrine progenitor stage and initiated expression of a β cell TF profile but are arrested in development prior to gaining INS expression. Collectively, these results indicate that the NKX2.2 SD is required for the continued progression of β cell differentiation beyond the initial precursor state. Of note, NKX6.1^+^INS^−^ cells were not TUNEL^+^ at E18.5 but were largely absent from *Nkx2.2^SDmut/Cre^* neonates at P2, at which time *Nkx6.1* and *ChgA* transcript levels were also significantly reduced (Supplemental Fig. S4A–C).

### Residual INS^+^ cells in *Nkx2.2^SDmut/Cre^* mice lack key features of functional β cells

**Figure 4.**
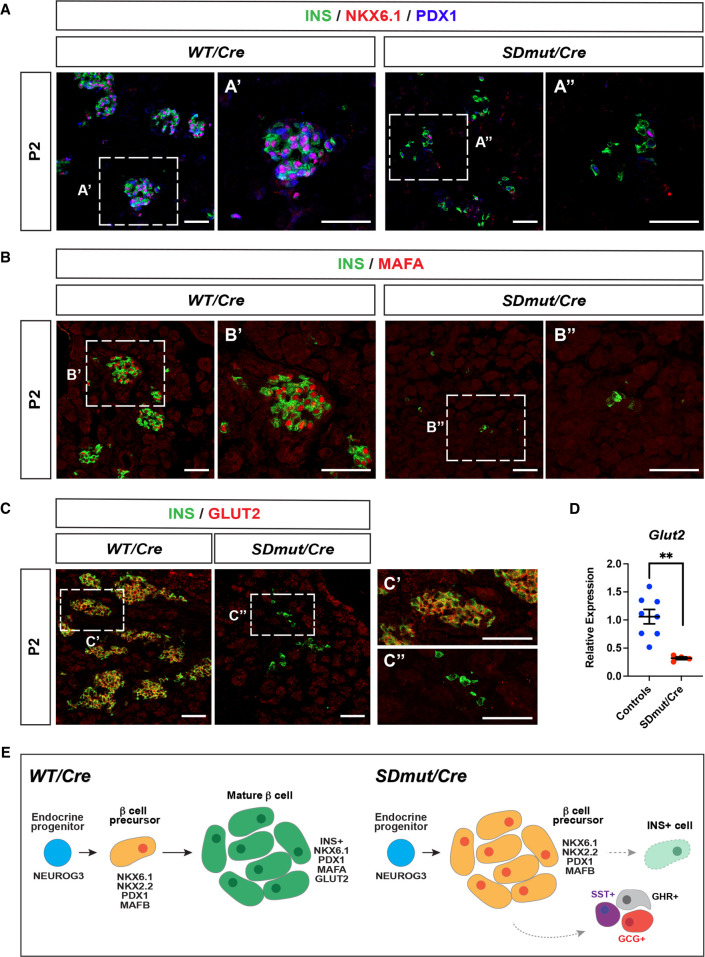
Residual INS^+^ cells in *Nkx2.2^SDmut/Cre^* mice lack features characteristic of functionally mature β cells. (*A*–*C*) The majority of the remaining INS^+^ cells in *Nkx2.2^SDmut/Cre^* (*SDmut/Cre*) animals do not exhibit NKX6.1 or PDX1 coexpression at P2 (shown in *A*). Similarly, MAFA (*B*) and GLUT2 (*C*) expression is also not observed. *A*, panel *A*′; *B*, panel *B*′; and *C*, panel *C*′ show higher magnification of *Nkx2.2^Cre/+^* (WT/*Cre*) images, while *A*, panel *A*′′; *B*, panel *B*′′; and *C*, panel *C*′′ show higher magnification of *Nkx2.2^SDmut/Cre^* staining. (*D*) qRT-PCR of *Glut2* in P2 pancreata. (*E*) Schematic summarizing changes in β cell development resulting from mutation of the SD. Data are presented as mean ± SEM. (**) *P* < 0.01. Scale bars, 50 µm.

Although *Nkx2.2^SDmut/Cre^* animals exhibited a significant decrease in β cell generation, this reduction in cell number alone does not account for the severe hyperglycemia observed in mutants at P2 (Fig. 1F; Peshavaria et al. 2006). We therefore examined the remaining INS^+^ cells in *Nkx2.2^SDmut/Cre^* neonates for expression of other factors regulating β cell performance. While residual INS^+^ cells in *Nkx2.2^SDmut/Cre^* mice sporadically retained expression of NKX6.1 and/or PDX1, expression of MAFA was barely detectable in these cells at E18.5 and was not recovered postnatally (Fig. 4A,B; Supplemental Fig. S5A,B). Furthermore, expression of GLUT2, the primary glucose transporter in β cells (Thorens 2015), was absent in P2 mutants, and qPCR analysis confirmed that transcript levels were significantly diminished (Fig. 4C,D). Collectively, these observations demonstrate that in addition to ensuring the progression of β cell precursor differentiation, the NKX2.2 SD further mediates β cell maturation (Fig. 4E).

### Nkx2.2 SD regulates β cell function in adult mice

**Figure 5.**
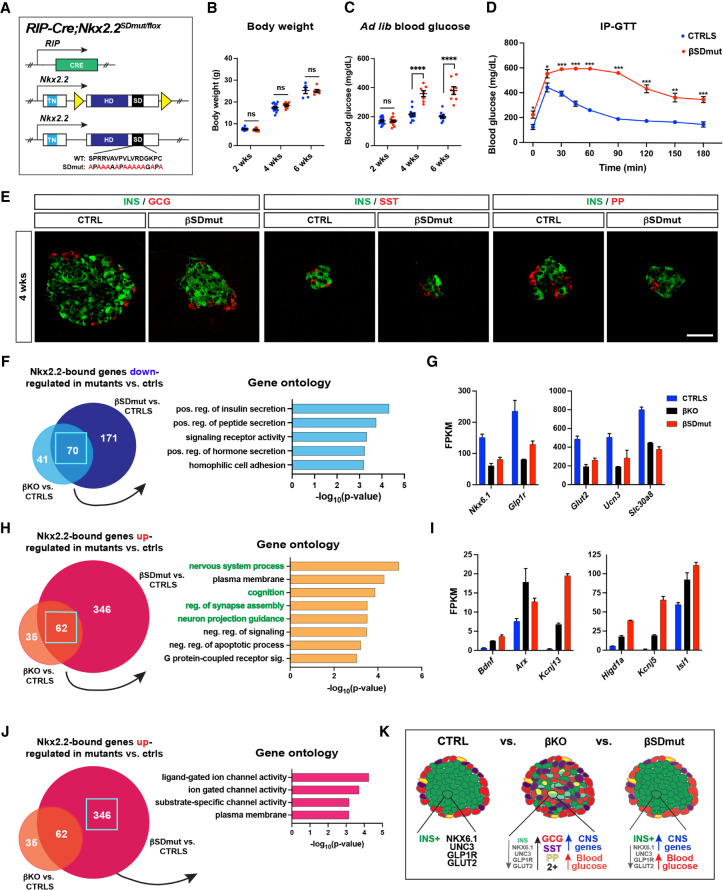
*RIP-Cre;Nkx2.2^SDmut/flox^* (βSDmut) animals develop diabetes due to impaired β cell function. (*A*) Schematic of alleles used to generate βSDmut mice. (*B*,*C*) Body weight (*B*) and ad lib blood glucose levels (*C*) in male βSDmut animals. (*D*) βSDmut males show elevated fasting blood glucose levels and impaired glucose clearance at 4 wk compared with littermate *RIP-Cre;Nkx2.2^flox/+^* control animals (CTRLS) during the intraperitoneal glucose tolerance test (IP-GTT). (*E*) INS, SST, and PP expression in 4-wk βSDmut animals. (*F*–*K*) RNA-seq analysis of NKX2.2-bound genes that are significantly altered in βSDmut islets versus CTRLS compared with NKX2.2-bound genes that are significantly altered in *RIP-Cre;Nkx2.2^flox/flox^* (βKO) animals versus CTRLS. (*F*) Gene ontology (GO) analysis of the 70 genes bound by NKX2.2 and down-regulated in both βSDmut and βKO islets compared with CTRL mice. (*G*) FPKM values of selected genes from *F*. (*H*) GO analysis of the 62 genes bound by NKX2.2 and up-regulated in both βSDmut and βKO versus CTRL animals. (*I*) FPKM values of selected genes from *H*. (*J*) GO analysis of the 346 transcripts bound by NKX2.2 and only up-regulated in βSDmut animals. (*K*) Schematic comparing differences in islet morphology and function in βKO versus βSDmut mice. Data are presented as mean ± SEM. (ns) Not significant, (*) *P* < 0.05, (**) *P* < 0.01, (***) *P* < 0.001. Scale bars represent 50 µm.

The lack of MAFA and GLUT2 expression in residual INS^+^ cells of *Nkx2.2^SDmut/Cre^* animals suggested that the SD may impact β cell function directly. In addition to its expression in pancreatic progenitors, NKX2.2 is also present in postmitotic β cells throughout adulthood. Our previous analysis of *RIP-Cre;Nkx2.2^flox/flox^* (βKO) mice demonstrated that NKX2.2 is required specifically within adult β cells to maintain INS content, regulate INS secretion, and prevent transdifferentiation and polyhormonal protein expression (Gutiérrez et al. 2017). To determine whether loss of the NKX2.2 SD similarly influences β cells, we generated *RIP-Cre;Nkx2.2^SDmut/flox^* (βSDmut) mice in which the wild-type *Nkx2.2^flox^* allele was removed by Cre recombinase in β cells only after initiation of INS expression, thereby allowing assessment of the function of the SD independently of its role in differentiation (Fig. 5A; Mastracci et al. 2013). RNA-seq analysis on βSDmut 8-wk islets confirmed that the *Nkx2.2^flox^* allele was deleted and only the *Nkx2.2^SDmut^* allele was expressed in the majority of β cells (Supplemental Fig. S6A,B).

Similar to βKO animals, both male and female βSDmut mice showed no differences in body weight compared with control (*RIP-Cre;Nkx2.2^flox/+^*) animals but became hyperglycemic and glucose intolerant at 4 wk of age (Fig. 5B–D; Supplemental Fig. S6C–E). However, unlike NKX2.2 βKO islets, βSDmut pancreata did not show decreased INS expression and/or coexpression of INS with GCG, SST, pancreatic polypeptide (PP), or gastrin (GAST) hormones (Fig. 5E; Supplemental Fig. S6F). Islet transcriptome analysis confirmed that hormone expression was comparable with control animals and that *Nkx2.2* transcript levels were not altered by mutation of the SD (Supplemental Fig. S6G).

While β cell hormonal identity was not affected in βSDmut mice, several genes influencing β cell function were significantly dysregulated (Supplemental Fig. S6H). To elucidate which of these transcripts were potentially controlled by the SD directly, we used our previously published NKX2.2 ChIP-seq to identify dysregulated genes in the βSDmut islets with NKX2.2 binding sites in their vicinity (Gutiérrez et al. 2017). We then compared these transcripts with NKX2.2-bound genes dysregulated in βKO islets (Fig. 5F–J; Supplemental Fig. S6I,J; Gutiérrez et al. 2017). Seventy genes were significantly decreased in both mutant animals, and gene ontology (GO) analysis showed that this shared subset of transcripts was most significantly enriched for genes associated with INS secretion, including *Nkx6.1*, *Glut2*, *Glp1r*, and *Unc3* (Fig. 5F,G). Together, these results indicate that the SD may mediate the role of NKX2.2 in regulating insulin secretory pathways (Gutiérrez et al. 2017).

To assess how the SD may contribute to NKX2.2 repressive activity, we next examined NKX2.2-bound genes that exhibited increased expression in both mutant islets (Fig. 5H,I). Surprisingly, GO analysis of the 62 targets derepressed in both βKO and βSDmut contexts showed substantial enrichment for terms related to neural development and function, suggesting that the SD may facilitate NKX2.2-dependent suppression of neural-related transcripts within the β cell (Fig. 5H). Analysis of NKX2.2-bound genes up-regulated only in βSDmut islets further revealed that many of the transcripts within this cohort are involved in ion channel activity and are also expressed within the nervous system (Fig. 5J). Collectively, these data suggest that in addition to ensuring the expression of genes necessary for β cell performance, the SD may also maintain silencing of neuron-related transcripts likely incongruent with β cell functionality (Fig. 5K).

### The SD may mediate interactions with members of the cohesion complex, chromatin modifiers, and the nuclear pore complex in β cells

To ascertain potential underlying mechanisms by which the SD contributes to NKX2.2-mediated regulation of gene expression, we performed mass spectrometry in MIN6 cells transfected with N-terminal MYC-tagged wild-type NKX2.2, TN mutant NKX2.2, SD mutant NKX2.2, or TN/SD mutant expression constructs (Supplemental Table S3). This analysis determined that mutation of the SD resulted in partial disruption of interactions with members of the cohesin complex and several chromatin modifiers (Supplemental Fig. S7A,B). Mutation of the SD also led to decreased interaction with numerous components of the nuclear pore complex (NPC), including NUP93, NUP210, MYO1C, and RANBP2 (Supplemental Fig. S7A,B).

### Generation of Nkx2.2-dependent CNS populations is not dependent on the SD

**Figure 6.**
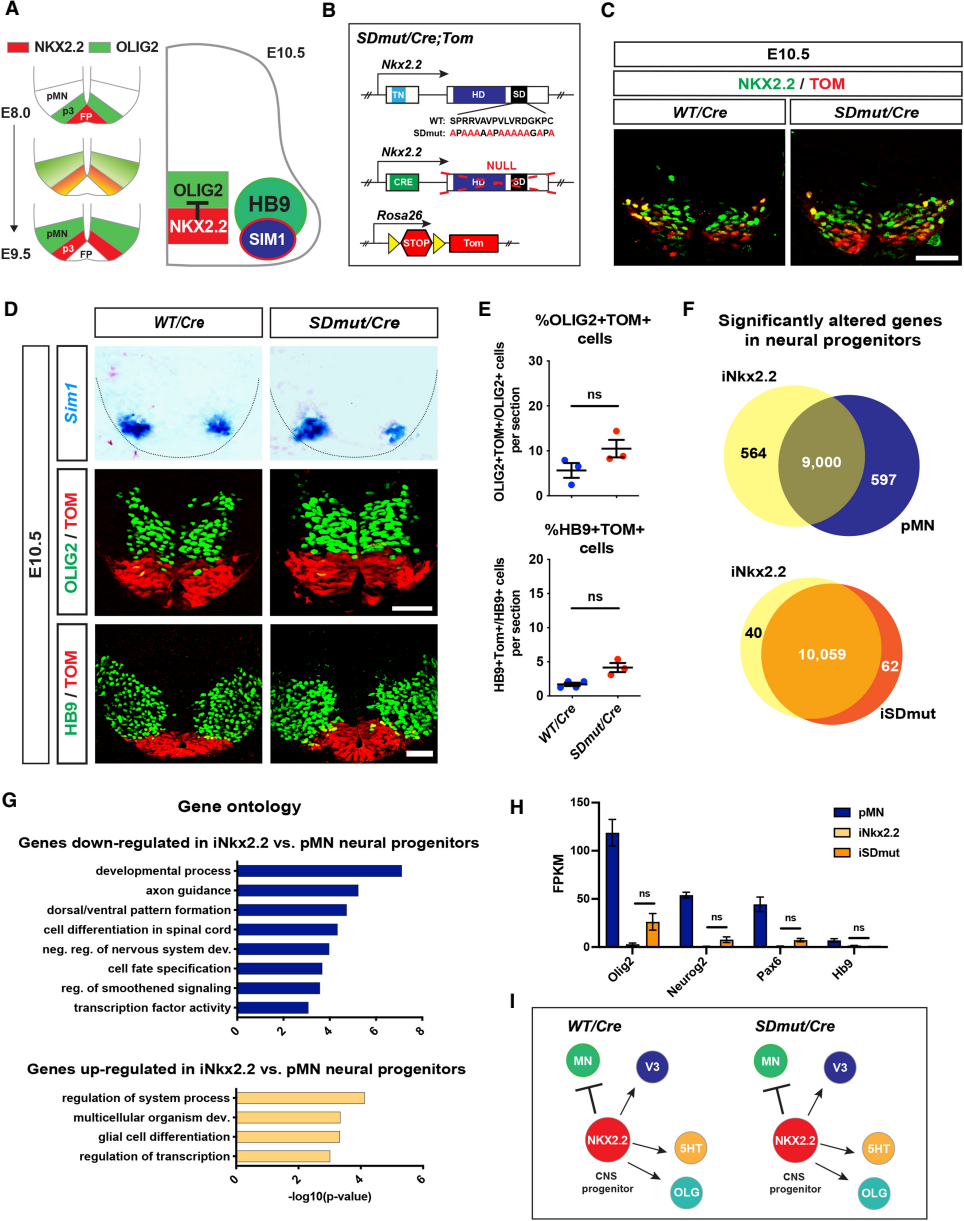
NKX2.2-dependent CNS populations are not affected by mutation of the SD. (*A*) Schematic representing the onset of NKX2.2-mediated repression of motor neuron (MN) identity. (*B*) Diagram of NKX2.2 alleles used to generate *Nkx2.2^SDmut/Cre^* (*SDmut/Cre*) and *Nkx2.2^Cre/+^* (WT/*Cre*) mice. The *Rosa26-Tomato* reporter allele (TOM) was included to enable lineage tracing. (*C*) NKX2.2 and TOM expression in E10.5 embryos. (*D*) In situ hybridization of *Sim1*, as well as OLIG2 and HB9 immunostaining, in *Nkx2.2^SDmut/Cre^* and *Nkx2.2^Cre/+^* E10.5 spinal cords. (*E*) Quantification of the percentage of OLIG2^+^TOM^+^ and HB9^+^TOM^+^ cells from *D*. (*F*) RNA-seq analysis of mouse embryonic stem cells (ESCs) differentiated into control motor neuron progenitors (pMN) and pMN progenitors in which either wild-type (iNKX2.2) or SD mutant (iSDmut) NKX2.2 was ectopically induced. (*G*) Gene ontology (GO) analysis of transcripts differentially expressed in iNKX2.2 progenitors compared with pMN cells. (*H*) FPKM values of TFs necessary for MN development. (*I*) Schematic showing that all examined NKX2.2-dependent CNS populations (see Supplemental Fig. S8) are not affected by mutation of the SD. Data are presented as mean ± SEM. (ns) Not significant. Scale bars, 50 µm.

The striking pancreatic phenotypes in *Nkx2.2^SDmut/Cre^* and βSDmut animals led us to examine whether mutation of the SD caused similar defects within the CNS. Unlike the pancreas, where NKX2.2 is expressed in pancreatic progenitors that give rise to all endocrine and exocrine cell types, NKX2.2 expression in the developing neuroepithelium is confined to defined subsets of progenitor populations (Fig. 6A; Sussel et al. 1998; Briscoe et al. 1999; Qi et al. 2001; Balderes et al. 2013). We therefore incorporated the *R26R-Tomato* (TOM) reporter to enable lineage tracing of wild-type versus SD mutant NKX2.2-derived CNS cell types (Fig. 6B; Madisen et al. 2010). NKX2.2 expression was faithfully recapitulated by the TOM reporter and appeared similar between *Nkx2.2^Cre/+^* and *Nkx2.2^SDmut/Cre^* embryos (Fig. 6C).

We first examined the role of the SD in three well-characterized CNS populations dependent on NKX2.2 function: V3 interneurons (INs), serotonergic (5HT) neurons, and oligodendrocytes (OLGs). In *Nkx2.2*-null mice, V3 and 5HT neurons are greatly reduced, and the maturation of OLGs is delayed (Briscoe et al. 1999; Qi et al. 2001; Zhu et al. 2014). Previous work has indicated that the NKX2.2 TN domain substantially influences NKX2.2 performance within these cell types, but the function of the SD in the mammalian nervous system has not been explored (Muhr et al. 2001; Zhang et al. 2020). Surprisingly, *Nkx2.2^SDmut/Cre^* animals did not exhibit any apparent anomalies in the differentiation of these subpopulations. Generation of *Sim1*^+^ V3 INs, 5HT^+^ neurons, and mature OLGs was comparable between *Nkx2.2^Cre/+^* and *Nkx2.2^SDmut/Cre^* rodents at several stages of development (Fig. 6D; Supplemental Figs. S8A–C, S9A–D). However, the early postnatal lethality associated with the mutant animals precluded assessment of any long-term functional defects.

### Nkx2.2-mediated repression of MN identity is not dependent on the SD

This disparity in pancreas versus CNS phenotypes in *Nkx2.2^SDmut/Cre^* mice was unexpected, as NKX2.2 functions similarly to regulate cell lineage decisions within these two systems (Briscoe et al. 1999; Prado et al. 2004). We therefore examined *Nkx2.2^SDmut/Cre^* animals for abnormalities in NKX2.2-dependent suppression of MN differentiation that may have occurred independently of V3 generation. V3 INs are specified from progenitors located within an area of the ventral neural tube, termed the p3 domain (Fig. 6A; Briscoe et al. 1999). Prior to NKX2.2 expression in this region, however, p3 progenitors express the MN determinant OLIG2. V3 identity is only established in the p3 domain once NKX2.2 becomes induced in OLIG2^+^ p3 progenitors, as NKX2.2 expression represses OLIG2 and thereby inhibits further generation of MNs from this region (Fig. 6A; Briscoe et al. 1999; Novitch et al. 2001; Ribes et al. 2010).

To elucidate whether the SD influences NKX2.2-mediated suppression of MN cell fate, we used the TOM reporter to examine whether the NKX2.2-derived V3 INs and progenitors exhibited coexpression of MN markers in *Nkx2.2^SDmut/Cre^* embryos. We assessed TOM^+^ cells at E10.5, when MN generation has normally been abrogated by NKX2.2, and V3 identity has been well established. However, no statistical differences in quantification of OLIG2^+^TOM^+^ or HB9^+^TOM^+^ cells were found between *Nkx2.2^Cre/+^* and *Nkx2.2^SDmut/Cre^* mice, indicating that within the spinal cord, the SD does not mediate NKX2.2 repressive activity (Fig. 6D,E).

### Transcriptome analysis reveals few, if any, differences between WT and SD mutant NKX2.2-expressing neural progenitors

Although we did not detect any overt phenotypic changes in the CNS of *Nkx2.2^SDmut/Cre^* mice, the limitations associated with obtaining sufficient cell numbers from embryonic tissue hindered molecular analysis that could reveal more subtle differences. We therefore generated mouse embryonic stem cells (ESCs) that possessed either a wild-type (iNkx2.2) or our SD mutated (iSDmut) allele of NKX2.2 tagged by FLAG and under the control of a doxycycline (DOX)-inducible promoter (Supplemental Fig. S10A; Mazzoni et al. 2011). These cell lines were differentiated toward the MN cell fate using a well-established ESC-to-MN differentiation protocol that generates OLIG2^+^ MN progenitors as well as HB9^+^ postmitotic MNs (Supplemental Fig. S10A; Wichterle et al. 2002). To recapitulate in vivo NKX2.2-mediated suppression of MN cell fate within the p3 domain, we added DOX to nascent OLIG2^+^ cultures in order to express NKX2.2 in these early MN progenitors (Supplemental Fig. S10A–C). *Nkx2.2* expression was activated to a similar extent in both iNKX2.2 and iSDmut embryoid bodies (Supplemental Fig. S10D), and expression of SD mutant NKX2.2 did not induce transcription of the endogenous wild-type *Nkx2.2* allele in iSDmut cells (Supplemental Fig. S6A,B). In agreement with the NKX2.2-mediated repression of MN identity observed in *Nkx2.2^Cre/+^* and *Nkx2.2^SDmut/Cre^* embryos, DOX induction of either wild-type or SD mutant NKX2.2 silenced both OLIG2 and HB9 expression during the differentiation (Supplemental Fig. S10E). We next performed global expression analysis on these DOX-induced iNkx2.2 and iSDmut neural progenitors and compared them with uninduced OLIG2^+^ MN progenitors (pMNs) as a control. Assessment of iNkx2.2 and pMN transcriptional profiles revealed >1000 significantly altered genes that were highly enriched for developmentally relevant GO terms (Fig. 6F,G). In contrast, only 102 genes were differentially expressed between iNKX2.2 and iSDmut progenitors, and no developmentally relevant GO terms were identified within these transcripts (Fig. 6F), indicating that both wild-type and SD mutant NKX2.2 efficiently silenced the MN transcriptional program with few, if any, meaningful differences. Indeed, mRNA levels of several factors critical for the establishment of MN identity were equally and efficiently repressed in both cell types (Fig. 6H). Collectively, these data suggest that unlike the pancreas, the SD is dispensable for CNS development (Fig. 6I).

## Discussion

In this study, we demonstrate that the cell-specific functions of NKX2.2 in the pancreas versus the CNS are driven by the highly conserved NKX2.2 SD. Our results indicate that the SD is required to drive the differentiation of nascent β cell precursors into functionally mature β cells in the embryonic pancreas and regulate genetic networks controlling β cell performance in the adult islet. Mutation of the SD during either stage of development led to the onset of severe diabetes. In stark contrast, the SD appeared expendable for the generation of all examined NKX2.2-dependent CNS populations. The nervous system and pancreas share expression of an overabundance of TFs, and to our knowledge, no other known mutations within this common set of proteins have resulted in phenotypic anomalies arising in only one of the two tissues.

Unlike other *Nkx2.2* mutant animals, the loss of β cells in *Nkx2.2^SDmut/Cre^* embryos was not entirely compensated for by the expansion of other endocrine lineages (Sussel et al. 1998; Prado et al. 2004; Papizan et al. 2011; Balderes et al. 2013; Churchill et al. 2017). Instead, a large portion of fated β cells stalled in differentiation after exiting the NEUROG3^+^ progenitor stage but prior to gaining INS expression. Although these arrested β precursors lacked INS, they expressed NKX2.2, NKX6.1, PDX1, and MAFB—TFs integral to β cell maturation and/or INS production (Sussel et al. 1998; Sander et al. 2000b; Artner et al. 2007; Gao et al. 2014). Together, these observations imply that the NKX2.2 SD may not only mediate repression of alternative lineages but also regulate the progression of β cell differentiation in part by impeding the function of one or more of these transcriptional regulators.

Consistent with this observation, both *MafB* and *Nkx6.1* mutant mice share several phenotypic similarities with *Nkx2.2^SDmut/Cre^* animals. Analogous to *Nkx2.2^SDmut/Cre^* embryos, *Neurog3-Cre;Nkx6.1^flox/−^* mice exhibit decreased β cell generation that is accompanied by a seemingly commensurate expansion of all other examined endocrine populations (Schaffer et al. 2013). In *MafB^−/−^* animals, β cell maturation is impeded, but embryonic expression of NKX6.1, PDX1, and NKX2.2 remains normal despite a lack of INS, MAFA, or GLUT2 expression (Artner et al. 2007). Unlike *Nkx2.2* and *Neurog3-Cre;Nkx6.1^flox/−^* mutant animals, however, the decreased production of β cells in the *MafB*-null is not accompanied by expansion of other endocrine populations (Artner et al. 2007). This is consistent with the function of MAFB as a well-known activator of β cell genes, rather than as a repressor of non-β cell programs (Kataoka et al. 1994; Matsuoka et al. 2003; Artner et al. 2006, 2007). These data suggest that the NKX2.2 SD may enable NKX6.1 and/or MAFB activity in β precursors by directly facilitating their repressor and activator functions, respectively, although the exact mechanisms remain to be identified.

Within the β cell itself, mutation of the SD resulted in early-onset diabetes that was comparable in severity with that observed in βKO animals (Gutiérrez et al. 2017). However, in contrast to βKO mice, βSDmut islets did not show reduced INS content, undergo cell fate conversion, or exhibit polyhormonal gene expression, indicating that the SD mediates the role of NKX2.2 in maintaining β cell utility independent of INS expression or the preservation of monohormonal identity. Genes involved in INS secretion, however, were greatly reduced in both mutant animals, demonstrating that NKX2.2 may use the SD to directly activate this specific gene cohort. In terms of repressive activity, targets up-regulated in both mutant animals were surprisingly enriched for neuronal genes, suggesting that the SD may also regulate NKX2.2-mediated suppression of neural transcriptional networks.

Our findings within the nervous system indicated that mutation of the SD did not affect the generation of NKX2.2-dependent cell types, although we cannot exclude the possibility that subtle phenotypes arose in mutant mice that we were unable to detect. In 5HT and OLG populations, however, NKX2.2 is not expressed postmitotically (Briscoe et al. 1999; Qi et al. 2001; Fu et al. 2002). Loss of the SD is thus unlikely to affect the function of mature 5HT neurons and OLGs. While NKX2.2 has been detected in postmitotic V3 INs at E12.5, expression beyond this stage of development has not been explored, and the function of NKX2.2, if any, within V3 neurons is unknown (Deska-Gauthier et al. 2020). Last, it remains possible that both neural and OLG subtype specifications were slightly altered as a result of SD mutation. The early postnatal lethality associated with the *Nkx2.2^SDmut/Cre^* mice precluded our ability to assess the identity and functionality of NKX2.2-derived CNS populations. Future studies analyzing mice in which the SD is mutated specifically within the CNS would address these concerns.

Although the SD region is highly conserved and is the defining feature of the NK2 family, little is known about its molecular activity. Characterization of the SD in the *Drosophila* homolog Vnd suggested that the SD stabilizes the interaction between Vnd and the Groucho corepressor, yet the SD deletion only moderately affected Vnd repressive activity in luciferase assays and in vivo analyses (Uhler et al. 2007). Furthermore, the *Nkx2.2^SDmut/Cre^* mice display a phenotype distinct from that of mice lacking the GRG-interacting TN domain, and mutations of the SD versus TN domain appear to disrupt interactions with different molecular complexes (Papizan et al. 2011; Zhang et al. 2020). On the other hand, in vitro experiments using overexpression of human *Nkx2.2* constructs suggested that deletion of the SD unmasked a C-terminal activation domain (Watada et al. 2000). However, our mass spectrometry analysis did not identify a gain of interactions between the mutated SD and coactivator proteins. Nonetheless, it remains possible that the SD is involved in regulating other intramolecular interactions.

Interestingly, the mass spectrometry experiments did uncover potential interactions between NKX2.2 and several members of the NPC that were reduced by mutation of the SD. Consistent with the RNA-seq analysis that revealed both down-regulated and up-regulated SD-dependent transcripts, NPC proteins have been shown to mediate both activating and repressive activities, such as enhancer regulation, TF complex assembly, chromatin remodeling and modification, and maintenance of H3K9me3-associated domains (Pascual-Garcia and Capelson 2021). Interactions between NKX2.2 and many cofactors involved in these gene-regulatory processes, including EHMT2, ATRX, SMC5/6, SMC3, and RAD21, were also found to be specifically dependent on the SD. Together, these results suggest that the SD may control both transcriptional activation and repression by directing NKX2.2-bound targets to the NPC and/or facilitating the interaction between these genes and chromatin regulators located within the NPC region. Future studies will address these possibilities.

In conclusion, we have identified the SD as a key factor distinguishing NKX2.2 functions in the pancreas versus the CNS. These studies highlight the need to dissect the cell-specific functions of TFs in order to uncover their heretofore unknown roles in lineage specification and maintenance. Interestingly, current in vitro protocols for differentiating human pluripotent stem cells into β cells yield large numbers of immature and precursor β cells, similar to the arrested populations seen in the *Nkx2.2^SDmut^* mice (Hrvatin et al. 2014; Russell et al. 2020). Understanding the more nuanced molecular mechanisms determining tissue-specific TF functionality in the pancreas versus the CNS may therefore aid in improving the efficient derivation of human β cells in vitro.

## Materials and methods

### Generation of Nkx2.2^SDmut^ mice

*Nkx2.2^SDmut^* mice were created by mutating conserved amino acids within the endogenous *Nkx2.2* locus into alanine residues while leaving amino acids important for structural integrity intact (Fig. 1A,B). To create the *Nkx2.2^SDmut^* allele, overlap extension PCR was used to introduce a 99-bp synthetic peptide encoding the mutated SD and a unique SacII digestion site into the *Nkx2.2* locus. Correct incorporation of the mutation in the *Nkx2.2^SD^* targeting vector was verified by DNA sequencing. The *Nkx2.2^SDmut^* targeting vector was electroporated into embryonic stem cells (ESCs) containing the *Nkx2.2^LCA^* allele (Arnes et al. 2012). A two-step positive–negative selection was used to identify ESC clones that had undergone successful cassette exchange (Chen et al. 2011). Positive clones were verified by PCR and Southern blot analysis after digestion with SacII. *Nkx2.2^SDmut/+^* ES cells were injected into C57Bl/6 blastocysts and transferred to pseudopregnant C57Bl/6 mice. Male chimeras were bred with female C57Bl/6 mice. The FRT-flanked hygromycin cassette was removed by breeding to FlpE mice (Rodríguez et al. 2000). Animals were genotyped using the following PCR primers specific to the wild-type or *Nkx2.2^SDmut^* alleles: oDB90 (GTGTGGCAGTGCCGGTCTG), oDB91 (GCGGCAGCACCGGCAGCCGCA), and oDB92 (GACAACGTTAACGTTGGGATG) (Supplemental Fig. S1A). All methodologies used in the generation of the *Nkx2.2^SDmut^* mice were approved by the Vanderbilt University Animal Care and Use Committee.

### Animal maintenance

All mice were maintained as a heterozygous breeding colony on a C57Bl/6J background. Mice were housed and treated according to the Columbia University and Colorado University Institutional Animal Care and Use Committee approval protocols. Genotyping for *Nkx2.2^Cre/+^*, *Nkx2.2^flox/+^*, *RIP-Cre*, and *R26-Tom* has been previously described (Herrera 2000; Madisen et al. 2010; Balderes et al. 2013; Mastracci et al. 2013).

### Culture and generation of mouse ESC lines

Inducible ESC lines were cultured and generated as previously described (Mazzoni et al. 2011). Inducible lines created in this study include wild-type NKX2.2 (iNKX2.2) and SD mutant NKX2.2 (iSDmut), which harbors the same mutations as the *Nkx2.2^SDmut^* mice described above. iNKX2.2 and iSDmut ESC lines were differentiated into MNs as previously described (Wichterle et al. 2002). The first day of the differentiation protocol was referred to as day 0. To induce expression of the transgenes in OLIG2^+^ pMN, 1 µg/mL DOX was added late on day 3 for ∼16–24 h.

### Glucose tolerance test

Mice were fasted overnight for 12 h. Blood glucose measurements were then taken for the 0-min time point. The animals were next given an intraperitoneal injection of 20% glucose (2 mg/g body weight), and blood glucose levels were recorded at 15, 30, 45, 60, 90, 120, 150, and 180 min following intraperitoneal injection.

### Immunohistochemistry

A detailed description of immunostaining procedures is provided in the Supplemental Material. Briefly, sections were blocked in 2% normal donkey serum and then incubated with primary antibodies overnight at 4°C, followed by incubation with secondary antibodies for 2 h at room temperature. A list of all primary and secondary antibodies used is in Supplemental Table S1.

### In situ hybridization

*Sim1* expression was examined by in situ hybridization using a previously described probe (Fan et al. 1996). The probe was labeled with digoxigenin (DIG) using DIG RNA labeling mix (Roche 11277073910) following the manufacturer's instructions. The in situ hybridization procedure followed that outlined in Prado et al. (2004) with the following modifications: Ten-micrometer sections were postfixed for 1 h at room temperature, and Proteinase K treatment was omitted. Bound probes were visualized with BM Purple AP substrate (Roche 11442074001) and counterstained with Nuclear Fast Red (Thermo Fisher Scientific NC9483816).

### Image analysis

Bright-field images were obtained on a Leica DM5500 microscope and processed with Adobe Photoshop. Confocal images were acquired on a Zeiss LSM510 or LSM710 and processed with Fiji and Adobe Photoshop. Immunostained pancreata were counted manually in Fiji using every 10th section throughout the entire pancreas. The number of cells was normalized to total pancreas area as measured by DAPI staining and quantified in Fiji. For neural sections, three to four sections at least 150 µm apart were counted per animal.

### qRT-PCR analysis of embryonic and postnatal pancreas

Whole pancreata from E15.5, E18.8, and P2 animals were dissected on ice, placed into RLT buffer, homogenized, and flash-frozen in liquid nitrogen. RNA was extracted using the RNeasy mini kit (Qiagen 74104) according to the manufacturer's instructions. One microgram of total RNA and random hexamers were used to synthesize cDNA following the SuperScript III reverse transcriptase protocol (Thermo Fisher Scientific 18-091-050). qRT-PCR was performed using ∼100 ng of cDNA TaqMan probes (Thermo) and iTaq universal probes Supermix (Bio-Rad 1725131). Samples were normalized to β-actin. Probes are listed in Supplemental Table S2. Relative expression was determined using either fold change relative to control samples (E15.5–E18.5) or the 2^ΔΔCt^ method (P2). When not labeled as WT/Cre, controls represent a combination of *Nkx2.2^Cre/+^*, *Nkx2.2^SD/^*^+^, and *Nkx2.2^+/+^* animals.

### Statistical analysis

Counting and qRT-PCR data were analyzed in Prism using either unpaired Student's *t*-test or one-way ANOVA. Data represent means ± SEM; *n* ≥ 3 or are indicated by data points unless otherwise stated. *P* < 0.05 was considered significant.

### RNA-seq, ChIP-seq, and mass spectrometry analysis

Standard procedures were used for all genomic, proteomic, and transcriptional analyses; specific details are documented in the Supplemental Material. Data generated in this study have been deposited in GEO under accession number GSE226345. Raw data files for the NKX2.2 ChIP-seq and the 4-wk βKO and control RNA-seq samples can also be found in GEO under accession number GSE79725 (Gutiérrez et al. 2017).

## Supplementary Material

Supplemental Material
