# Supplementary material for: Major β cell-specific functions of NKX2.2 are mediated via the NK2-specific domain
Source: Genes Dev. 2023 Jun 1;37(11-12):490–504. doi: 10.1101/gad.350569.123 (PMC10393193; doi:10.1101/gad.350569.123)
Supplement: Supplemental Material [file supp_37_11-12_490__DC1.html]

Major β cell-specific functions of NKX2.2 are mediated via the NK2-specific domain — Major β cell-specific functions of NKX2.2 are mediated via the NK2-specific domain — Supplemental Material 

# Major β cell-specific functions of NKX2.2 are mediated via the NK2-specific domain

## Supplemental Material

- Supplemental\_Materials\_and\_Methods.docx
- Supplemental\_Fig\_S1.pdf
- Supplemental\_Fig\_S2.pdf
- Supplemental\_Fig\_S3.pdf
- Supplemental\_Fig\_S4.pdf
- Supplemental\_Fig\_S5.pdf
- Supplemental\_Fig\_S6.pdf
- Supplemental\_Fig\_S6\_legend.pdf
- Supplemental\_Fig\_S7.pdf
- Supplemental\_Fig\_S8.pdf
- Supplemental\_Fig\_S9.pdf
- Supplemental\_Fig\_S10.pdf
- Supplemental\_Fig\_S10\_legend.pdf
- Supplemental\_tables.pdf
