# Supplementary material for: Major β cell-specific functions of NKX2.2 are mediated via the NK2-specific domain
Source: Genes Dev. 2023 Jun 1;37(11-12):490–504. doi: 10.1101/gad.350569.123 (PMC10393193; doi:10.1101/gad.350569.123)
Supplement: Supplemental Material [file supp_gad.350569.123_Supplemental_Materials_and_Methods.docx]

**Supplemental Materials and Methods**

*Detailed immunohistochemistry procedures*

For embryonic and early postnatal pancreas, E12.5 whole embryos were fixed in 4% PFA for 4hrs at 4°C, washed in PBS at room temperature (RT), cryoprotected in 30% sucrose, and frozen in OCT (Tissue-Tek, NC9636948) using dry ice. E15.5 embryos were treated in the same manner, but the head and lower extremities were removed before fixation. E18.5 and P2 pancreata were dissected and fixed for 4hrs for all stainings except for NKX2.2 (which required a 2hr fixation). 10μm sections were blocked in 2% normal donkey serum diluted in PBS with 0.1% TritonX (NDS-PBST) for 30min at RT and then incubated in primary antibodies diluted in NDS-PBST overnight at 4°C. The next day, sections were stained with secondary antibodies diluted 1:500 in NDS-PBST for 2hrs at RT, followed by DAPI (1:1000 in PBS) for 10min at RT.

Adult pancreata were dissected at 4wks of age. Samples were fixed in 10% neutral-buffered formalin (VWR) at 4°C for 2-4hrs and then transferred to 70% ethanol. Samples were embedded in paraffin, sectioned at 5µm, and stored at RT. Prior to staining, sections were boiled for 20min in antigen retrieval solution (10mM sodium citrate pH 6.0). Sections were then returned to RT for 20min, washed in PBST, and blocked in NDS-PBST for 30min at RT. Primary and secondary antibodies and DAPI were applied as described above.

For embryonic neural tissue, E10.5 whole embryos were fixed for 1-1.5 hours at 4°C in 4% paraformaldehyde (PFA), washed in PBS at RT, and cryoprotected through a 5%-10%-15% sucrose gradient at 4°C. Dissected spinal cords were then embedded in OCT and flash frozen in liquid nitrogen. For postnatal spinal cords, P0 pups were perfused with 4% PFA. Spinal cords were dissected (leaving surrounding skeletal structure intact), post-fixed for 2hrs at 4°C, washed in PBS, cryoprotected in 30% sucrose overnight at 4°C, and embedded as above. 15μm sections were blocked with 10% fetal bovine serum diluted in PBS with 0.1% TritonX (10%FBS-PBST) for 10min at RT. Sections were then incubated in primary antibodies diluted in 2%FBS-PBST overnight at 4°C. Secondary antibodies were diluted 1:1000 in 2%FBS-PBST and applied to sections for 2hrs at RT.

Embryoid bodies were prepared as previously described (Wichterle et al., 2002). Briefly, cells were fixed in 4% PFA for 10min at 4°C, washed in PBS, cryoprotected in 30% sucrose at 4°C for 30-60min, embedded in OCT, and frozen on dry ice. Sections were stained as described for neural tissue.

*RNA-seq analysis of E15.5 pancreata*

Total RNA concentration and quality were measured using the Agilent Bioanalyzer 2100. Inclusion of samples required RNA integrity (RIN) values ≥ 8.0. Samples were sequenced using Illumina NovaSeq6000 at the Colorado University Cancer Genomics Core Facility. Reads were mapped to the mouse genome (mm10) using the RNA-seq alignment algorithm in the STAR (Spliced Transcripts Alignment to a Reference) software package (version 2.3.1) (Dobin et al., 2013). Differential expression was determined using DESeq2 (Love et al., 2014). Significantly altered genes were determined based on an adjusted p value < 0.05. n ≥ 3. Generation of the heatmap was performed by pheatmap (version 1.0.12).

*RNA extraction of βSDmut islets*

Adult 8wk mice were perfused through the common hepatic bile duct with 1mg/mL Collagenase P (Roche, 11213857001)/M199 medium (Sigma, M5017). Pancreata were dissected and incubated at 37°C for 16min. Samples were washed multiple times with 10% fetal bovine serum/M199. Histopaque (Sigma) and M199 were used to separate islets on a gradient, and islets were subsequently picked under a dissection microscope. Islets were disrupted via vortexing, and total RNA was isolated using RNeasy Micro Kit (Qiagen, 74104).

*RNA-seq analysis of βSDmut adult islets*

RNA and library preparation for βSDmut and control 8wk islets was performed as described above for E15.5 pancreata. RNA was sequenced using Illumina HiSeq2000 at the Columbia University Genome Center. Alignment of reads was performed as above. FPKM values (Fragments Per Kilobase of exon per Million fragments mapped) were calculated per individual gene using Cufflinks software (version 2.2.1) (Trapnell et al., 2013). In order to compare transcriptional differences in βSDmut and βKO animals, we took advantage of our previously published RNA-seq comparing control and βKO 4wk islets (Gutierrez et al., 2017). RNA reads from 4wk control and βKO animals were re-processed concurrently with our 8wk samples. Control animals (*RIP-Cre; Nkx2.2^flox/+^*) in our final RNA-seq analysis consisted of two 8wk samples and two 4wk samples.

Differential RNA expression across cohorts was assessed using EdgeR (version 3.20.9) and limma (version 3.34.9) software packages and closely followed the pipeline described in Law, et al (2016) (Law et al., 2014, Phipson et al., 2016, Ritchie et al., 2015, Robinson et al., 2010, Law et al., 2016). Specifically, raw RNA reads of all replicate samples in the control, βKO, and βSDmut groups were analyzed together, and all genes which did not have a count of 2 (βSDmut vs. CTRL analysis) or a count of 10 (βSDmut vs. βKO vs. CTRL analysis) in at least two samples were excluded. Data were normalized using the trimmed mean of M-values (TMM) method with the calcNormFactors function (Robinson and Oshlack, 2010) prior to determining significant differences based on adjusted p value < 0.05. Z-scores were generated from FPKM values and used to generate heatmaps using Clustered Image Maps miner (CIMminer) software (Weinstein et al., 1997). Significantly altered genes were manually sorted based on log2 fold-changes in expression between control and SD-mutant comparisons obtained from differential expression analyses.

*RNA-seq analysis of ESC-derived neural progenitors*

RNA was extracted from day 4 embryoid bodies using Trizol Reagent (Life Technologies, SC: 15596-018) in combination with RNeasy Mini Kit (Qiagen, 74104). Inclusion of samples required RNA integrity (RIN) values > 8.0 as determined with Agilent Bioanalyzer 2100. Library preparation, sequencing, genome alignment, FPKM estimation, and determination of differential expression was performed as described above for βSDmut islets. “pMN” control samples in this analysis refer to ESCs that harbor the WT *Nkx2.2* inducible transgene (iNKX2.2) but that were not exposed to DOX during differentiation and therefore never expressed NKX2.2.

*Quantification of percentage of allele expression*

To quantify the percentage of Nkx2.2SDmut expression, a custom genome and GTF file that only included the Nkx2.2 gene (one entry for the WT allele and one for the SD-mutated allele) was created. RNA-seq reads were then aligned to this custom genome and GTF file using STAR (ver.2.7.10b). Reads that aligned equally to unchanged portions of the Nkx2.2 genes were eliminated, and thus only unique fragments that aligned to either the WT or SD-mutated domain were counted and used to determine the percentage of each allele present in the samples.

*Gene ontology (GO) analysis*

For RNA-seq analysis, GO terms were identified with the Gene Ontology Enrichment Analysis and Visualization (GOrilla) system using the default settings associated with the ranked (for embryoid bodies) or unranked list option (for βSDmut samples) (Eden et al., 2009).

*Nkx2.2 ChIP-seq analysis*

Our previously published NKX2.2 ChIP-seq data obtained from MIN6 cells (Gutierrez et al., 2017) was re-analyzed as follows. All replicate samples were re-aligned using Bowtie (version 0.12.7) (Langmead et al., 2009) to the mm10 assembly of the mouse genome. Peak calling was carried out using the GEM algorithm (Guo et al., 2012) with the default settings. Read-count intensities for each replicate were obtained within +/- 500bp and +/-100bp of all GEM identified peaks. Read counts of individual replicates were then normalized to their respective sequencing library sizes, and only loci above a given threshold in both replicates (determined based on the 500bp read counts) were used for subsequent analysis. After filtering out these very low-occupied regions, ChIP-seq peaks within 500bp of each other were merged using Bedtools (version 2.17.0) (Quinlan and Hall, 2010). The locus with the highest 100bp read density was identified using Tidyverse (version 1.2.1) (Wickham H. et al, 2019) and utilized as the center of the newly merged peak. Read counts +/-500bp around these new sites were obtained again for both replicates and converted to counts per million (CPM) by normalizing to the total number of remaining read counts in each sample using EdgeR (version 3.20.9) and limma (version 3.34.9) software packages (Law et al., 2016, Robinson et al., 2010, Ritchie et al., 2015). CPM values were further quantile normalized across replicates to account for variations in ChIP-seq quality using the normalizeQuantiles function (Hansen et al., 2012). Normalized values were then averaged and log2-transformed. These log2(CPM) values were used to assess the amount of NKX2.2 occupancy at each site.

*Assignment of Nkx2.2 ChIP-seq peaks to genes*

To link NKX2.2 peaks with their associated gene, each NKX2.2 site was assigned to the nearest TSS of known mRNA genes (20,567 of annotated mm10 NCBI Build 38 RefSeq mRNA genes having complete coding start and end sequences*).* In instances where multiple TSS occurred for a given gene, the most 5’ TSS was utilized. In instances where there was more than one NKX2.2 peak per gene, the most highly occupied NKX2.2 locus was used as determined by the log2(CPM) values described above.

*Mass spectrometry*

MIN6 cells were transfected with pcDNA3:MYC-NKX2.2, pcDNA3:MYC-NKX2.2^TNmut^, pcDNA3:MYC-NKX2.2^SDmut^, pcDNA3:MYC-NKX2.2^TNmut,SDmut^ , or pcDNA3:MYC (CTRL) using Lipofectamine 3000 (Thermo, L3000015) according to the manufacturer’s instructions. Cells were harvested 48hrs post-transfection, and nuclear extracts were collected using the Active Motif Nuclear Complex Co-IP Kit (Active Motif, 54001). Total nuclear protein was immunoprecipitated with Pierce Anti-c-Myc Magnetic Beads (Thermo, 88843) incubated in 1X Low Stringency Buffer (Active Motif) overnight at 4°C. The following day, beads were washed 6X in Wash Buffer (500mM NaCl, 0.5% Tx, 50mM HEPES, 1mM EDTA, 5% Glycerol, and protease/phosphatase inhibitors) and 1X in Wash Buffer with a lower NaCl concentration (150mM) and without detergent. Remaining proteins bound to beads were identified at University of Colorado School of Medicine Proteomics Core Facility via mass spectrometry. Spectra counts of proteins whose interactions with NKX2.2 were reduced specifically by mutation of the SD domain can be found in Supplementary Table 3. Z-scores were generated from these spectra counts and used to generate a heatmap using Clustered Image Maps miner (CIMminer) software (Weinstein et al., 1997). Enriched Gene Ontology (GO) terms were identified using the Protein Analysis Through Evolutionary Relationships (PANTHER) Classification System with the default settings (Mi et al., 2013, Thomas et al., 2022).
