# Supplementary material for: Major β cell-specific functions of NKX2.2 are mediated via the NK2-specific domain
Source: Genes Dev. 2023 Jun 1;37(11-12):490–504. doi: 10.1101/gad.350569.123 (PMC10393193; doi:10.1101/gad.350569.123)
Supplement: Supplemental Material [file supp_gad.350569.123_Supplemental_Fig_S1.pdf]

Abarinov Supplemental Figure S1.

A

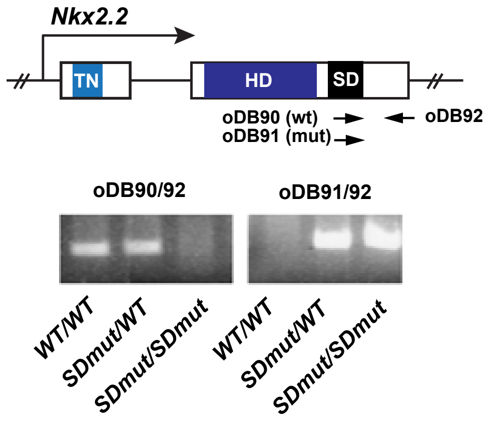

B

Animals at P2

Genotype	Observed	Expected
WT/Cre	10	9.75
SDmut/Cre	9	9.75
SDmut/WT	9	9.75
WT/WT	11	9.75

χ^2 p value = 0.96

C

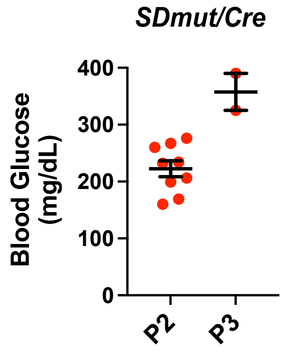

D

Animals at Weaning

Genotype	Observed	Expected
WT/Cre	9	7.75
SDmut/Cre	0	7.75
SDmut/WT	11	7.75
WT/WT	11	7.75

χ^2 p value = 0.01

Figure S1. Additional characterization of Nkx2.2$^{SDmut/Cre}$ mice. (A) Genotyping scheme for the Nkx2.2^{SDmut} allele. Gel shows the PCR products pertaining to Nkx2.2$^{+/+}$(WT/WT), Nkx-2.2$^{SDmut/+}$(SDmut/WT), and Nkx2.2$^{SDmut/SDmut}$ (SDmut/SDmut) animals. (B) Chi Square analysis at P2 demonstrates that Nkx2.2$^{SDmut/Cre}$ mice are born at normal Mendelian ratios. (C) Nkx2.2$^{SDmut/Cre}$ neonates experience worsening blood glucose levels at P3 compared to P2. Data are presented as mean ± SEM. (D) Chi Square analysis of genotypes obtained at weaning. No Nkx2.2$^{SDmut/Cre}$ animals survived to this point of development. n's are indicated in tables and by data points on graph.
