# Supplementary material for: Major β cell-specific functions of NKX2.2 are mediated via the NK2-specific domain
Source: Genes Dev. 2023 Jun 1;37(11-12):490–504. doi: 10.1101/gad.350569.123 (PMC10393193; doi:10.1101/gad.350569.123)
Supplement: Supplemental Material [file supp_gad.350569.123_Supplemental_Fig_S2.pdf]

Abarinov Figure S2.

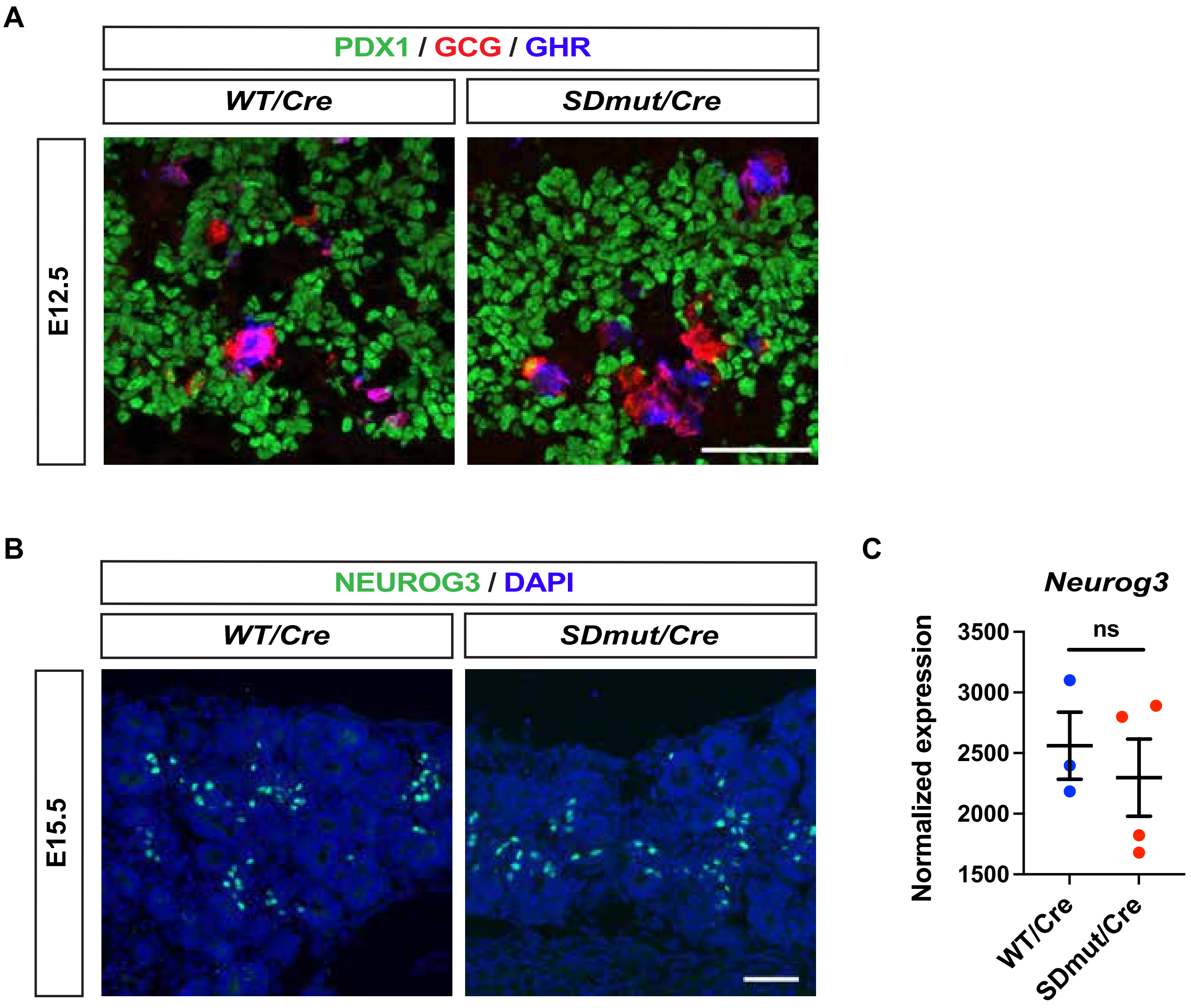

Figure S2. Endocrine specification at E12.5 and endocrine progenitor generation at E15.5 appear normal in Nkx2.2$^{SDmut/Cre}$ animals. (A) PDX1, GCG, and GHR expression is similar between Nkx-2.2$^{Cre/+}$ (WT/Cre) and Nkx2.2$^{SDmut/Cre}$ (SDmut/Cre) embryos at E12.5. n = 2. (B-C) NEUROG3 protein (B) and mRNA (C) expression are comparable between Nkx2.2$^{Cre/+}$ (WT/Cre) and Nkx2.2$^{SDmut/Cre}$ (SDmut/Cre) pancreata at E15.5. Transcript levels of Neurog3 were obtained from the RNA-seq described in Figure 3. n = 3-4. Data are presented as mean ± SEM. ns, not significant. Scale bars represent 50μm.
