# Supplementary material for: Major β cell-specific functions of NKX2.2 are mediated via the NK2-specific domain
Source: Genes Dev. 2023 Jun 1;37(11-12):490–504. doi: 10.1101/gad.350569.123 (PMC10393193; doi:10.1101/gad.350569.123)
Supplement: Supplemental Material [file supp_gad.350569.123_Supplemental_Fig_S3.pdf]

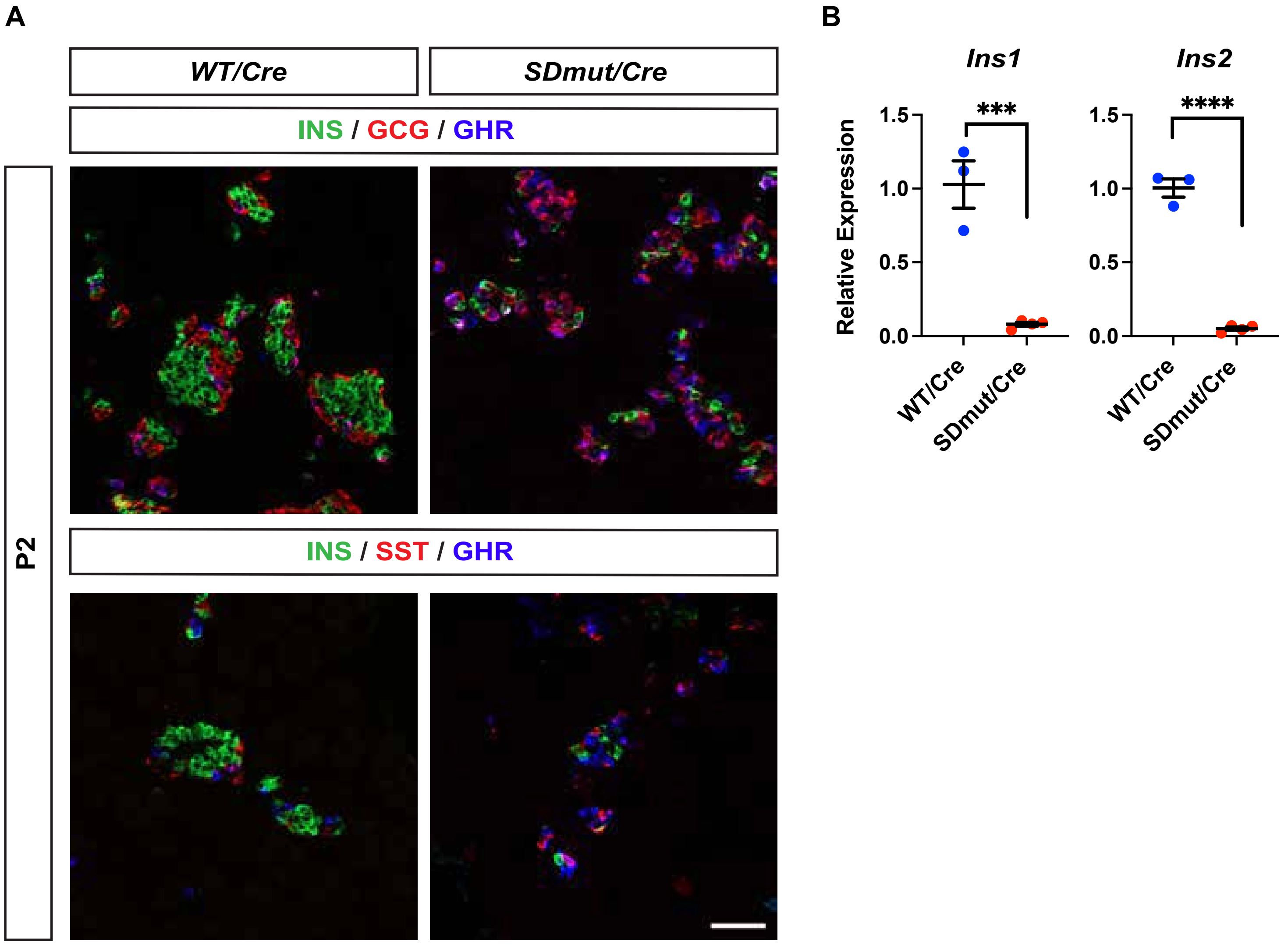

Figure S3. Nkx2.2$^{SDmut/Cre}$ neonates continue to show a loss of β cells and altered islet formation at P2. (A) Nkx-2.2$^{SDmut/Cre}$ mutant islets show reduced INS+ cells combined with an overrepresentation of GCG+, GHR+, and SST+ populations. (B) qRT-PCR analysis of Ins1 and Ins2 confirms the decreased β cell generation in Nkx2.2$^{SD-mut/Cre}$ animals. Data are presented as mean ± SEM. ***p<0.001, ****p<0.0001. n's are indicated by data points. Scale bars represent 50μm.
