# Supplementary material for: Major β cell-specific functions of NKX2.2 are mediated via the NK2-specific domain
Source: Genes Dev. 2023 Jun 1;37(11-12):490–504. doi: 10.1101/gad.350569.123 (PMC10393193; doi:10.1101/gad.350569.123)
Supplement: Supplemental Material [file supp_gad.350569.123_Supplemental_Fig_S4.pdf]

Abarinov Figure S4.

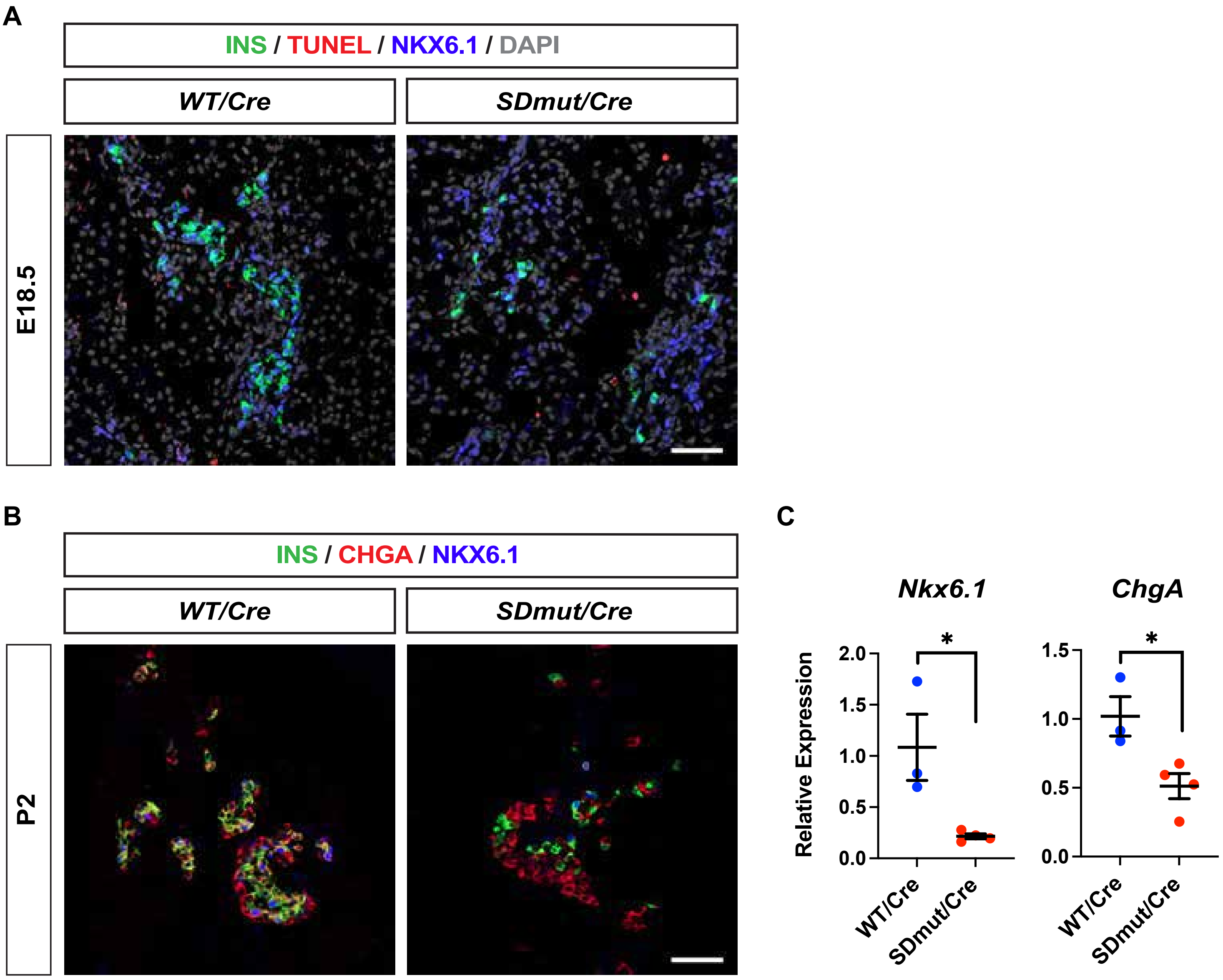

Figure S4. Stalled β cell precursors do not undergo apoptosis at E18.5 but are largely absent from P2 *Nkx2.2*$^{SD\text{-}mut/Cre}$ animals. (A) NKX6.1+INS- β precursors in *Nkx2.2*$^{SDmut/Cre}$ embryos are not TUNEL+ at E18.5. (B) Immunostaining at P2 demonstrates that NKX6.1 expression in *Nkx2.2*$^{SDmut/Cre}$ mice is mainly confined to INS+ cells. NKX-6.1+CHGA- β precursors are rarely visible. (C) *Nkx6.1* and *ChgA* transcript levels are decreased in P2 mutants. Data are presented as mean ± SEM. *$p<0.05$. n = 3-4. Scale bars represent 50μm.
