# Supplementary material for: Major β cell-specific functions of NKX2.2 are mediated via the NK2-specific domain
Source: Genes Dev. 2023 Jun 1;37(11-12):490–504. doi: 10.1101/gad.350569.123 (PMC10393193; doi:10.1101/gad.350569.123)
Supplement: Supplemental Material [file supp_gad.350569.123_Supplemental_Fig_S5.pdf]

Abarinov Figure S5.

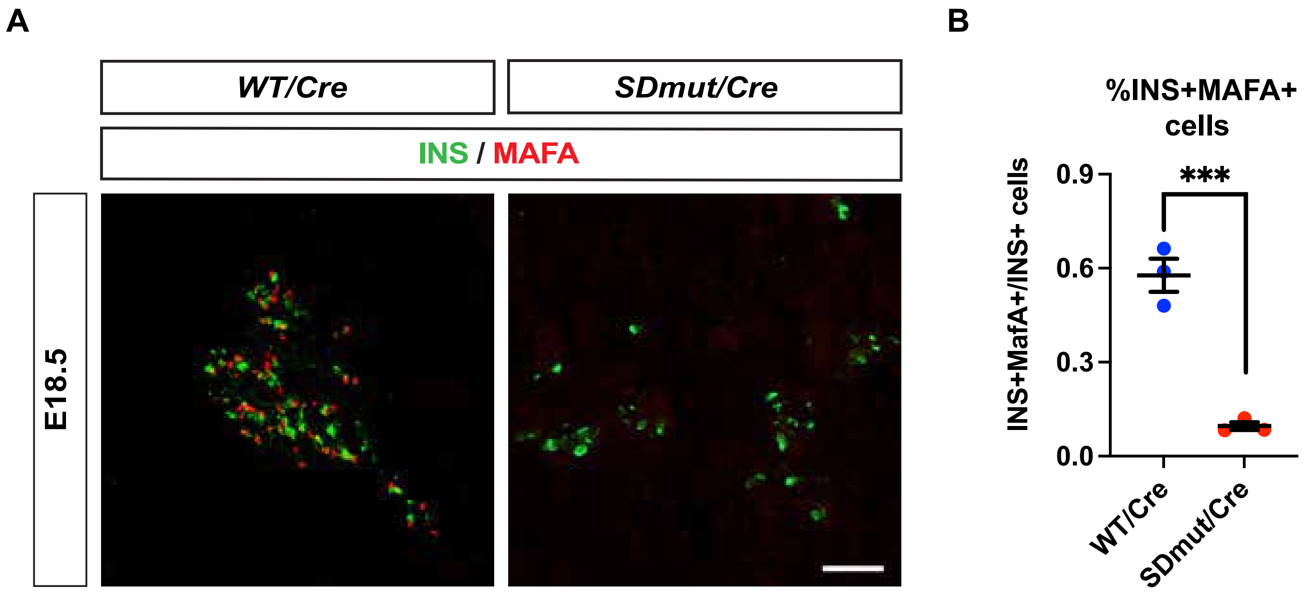

Figure S5. MAFA-expressing cells are significantly reduced in *Nkx2.2*$^{SDmut/Cre}$ embryos at E18.5. (A) INS and MAFA co-expression is rarely seen in *Nkx2.2*$^{SDmut/Cre}$ embryos. (B) Quantification confirms that the percentage of INS+ cells expressing MAFA is significantly reduced in *Nkx2.2*$^{SD-mut/Cre}$ mice. Data are presented as mean ± SEM. ***p<0.001. n's are indicated by data points. Scale bar represents 50µm.
