# Supplementary figures and images for: Major β cell-specific functions of NKX2.2 are mediated via the NK2-specific domain
Source: Genes Dev. 2023 Jun 1;37(11-12):490–504. doi: 10.1101/gad.350569.123 (PMC10393193; doi:10.1101/gad.350569.123)

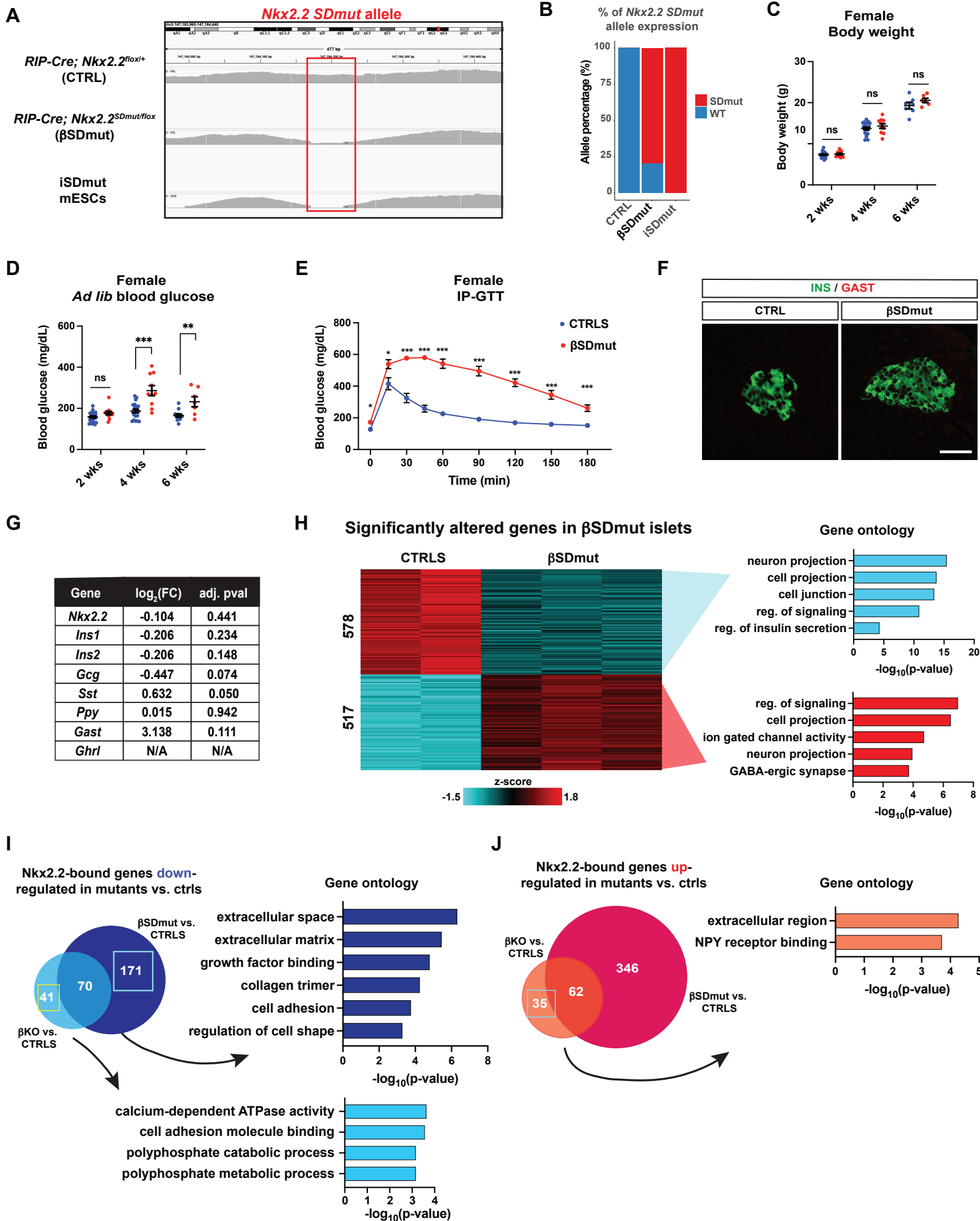

Supplement: Supplemental Material [file supp_gad.350569.123_Supplemental_Fig_S6.pdf]

A

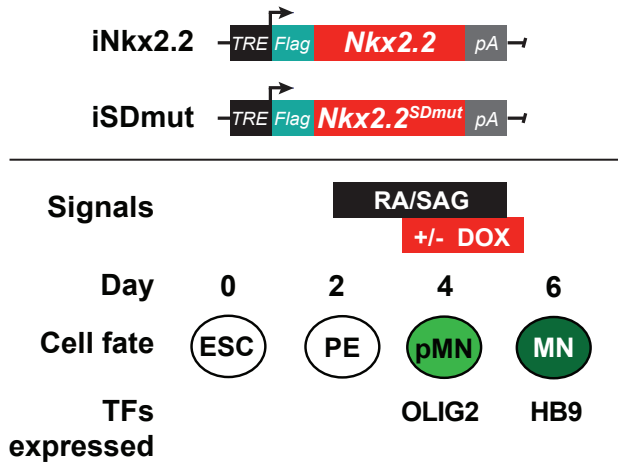

B

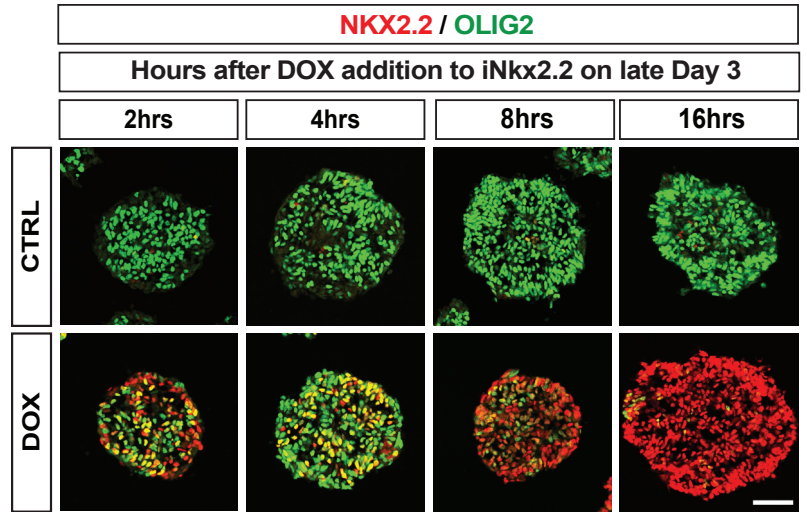

C

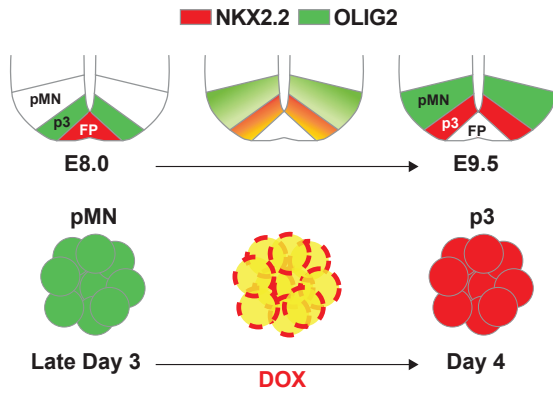

D

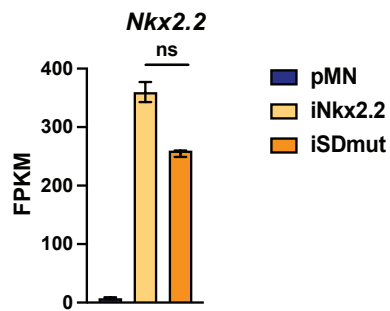

E

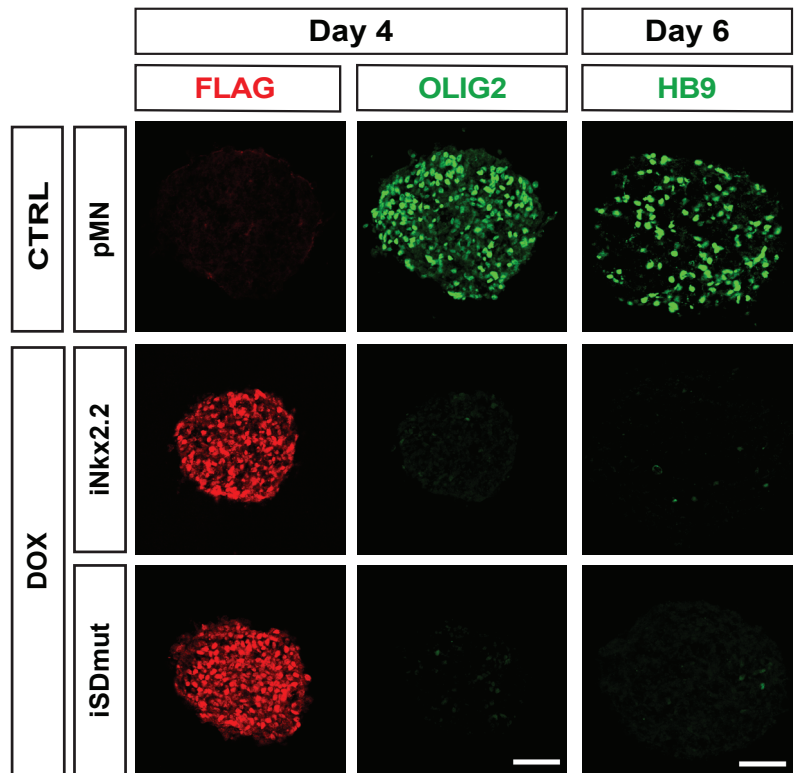

Supplement: Supplemental Material [file supp_gad.350569.123_Supplemental_Fig_S10.pdf]
