# Supplementary material for: Major β cell-specific functions of NKX2.2 are mediated via the NK2-specific domain
Source: Genes Dev. 2023 Jun 1;37(11-12):490–504. doi: 10.1101/gad.350569.123 (PMC10393193; doi:10.1101/gad.350569.123)
Supplement: Supplemental Material [file supp_gad.350569.123_Supplemental_Fig_S6_legend.pdf]

Abarinov Figure S6 Cont.

Figure S6. Further characterization of *RIP-Cre; Nkx2.2*$^{SDmut/flox}$ (βSDmut) animals. (A) Integrative Genomics Viewer (IGV) RNA-seq tracks from representative *RIP-Cre; Nkx2.2*$^{flox/+}$ (CTRL) and *RIP-Cre; Nkx2.2*$^{SDmut/flox}$ (βSDmut) adult islets and from iSDmut day 4 neural progenitors (see Fig. 6) at the endogenous wildtype (WT) *Nkx2.2* gene locus. RNA-seq reads from CTRL animals align to the SD domain (highlighted in red) while those from βSDmut and iSDmut samples do not—confirming expression of the mutant, rather than WT, SD allele in βSDmut mice and iSDmut cells. (B) Quantification of the percent expression of each *Nkx2.2* allele from RNA-seq analysis on adult islets. The small percentage of WT *Nkx2.2* expression in βSDmut animals is likely due to *Nkx2.2* expression that is maintained in α cells and/or incomplete deletion of the conditional *Nkx2.2*^{flox} allele in β cells. (C-E) Female βSDmut mice develop diabetes similar to males. Females exhibit normal body weight (C) but increased *ad lib* blood glucose levels (D) and impaired glucose clearance (E) during the intraperitoneal glucose tolerance test (IP-GTT) compared to control *RIP-Cre; Nkx2.2*$^{flox/+}$ (CTRL) animals at 4wks. (F) Gastrin (GAST) protein expression is absent from both CTRL and βSDmut mice at 4wks. (G) RNA-seq analysis of islets from CTRL and βSDmut 8wk animals shows no significant changes in *Nkx2.2* or hormone expression. (H) Heatmap (left) and gene ontology (GO) analysis (right) of significantly altered genes identified between βSDmut and CTRL 8wk islets. (I) GO analysis of the 171 genes bound by NKX2.2 and specifically down-regulated in βSDmut animals compared to CTRLS and of the forty-one genes specifically down-regulated in βKO vs. CTRL islets. (J) GO analysis of the thirty-five genes specifically up-regulated in βKO vs. CTRL islets. Data are presented as mean ± SEM. *p<0.05, **p<0.01, ***p<0.001. n's are indicated by data points. Scale bar represents 50μm.
