# Supplementary material for: Major β cell-specific functions of NKX2.2 are mediated via the NK2-specific domain
Source: Genes Dev. 2023 Jun 1;37(11-12):490–504. doi: 10.1101/gad.350569.123 (PMC10393193; doi:10.1101/gad.350569.123)
Supplement: Supplemental Material [file supp_gad.350569.123_Supplemental_Fig_S7.pdf]

Abarinov Figure S7.

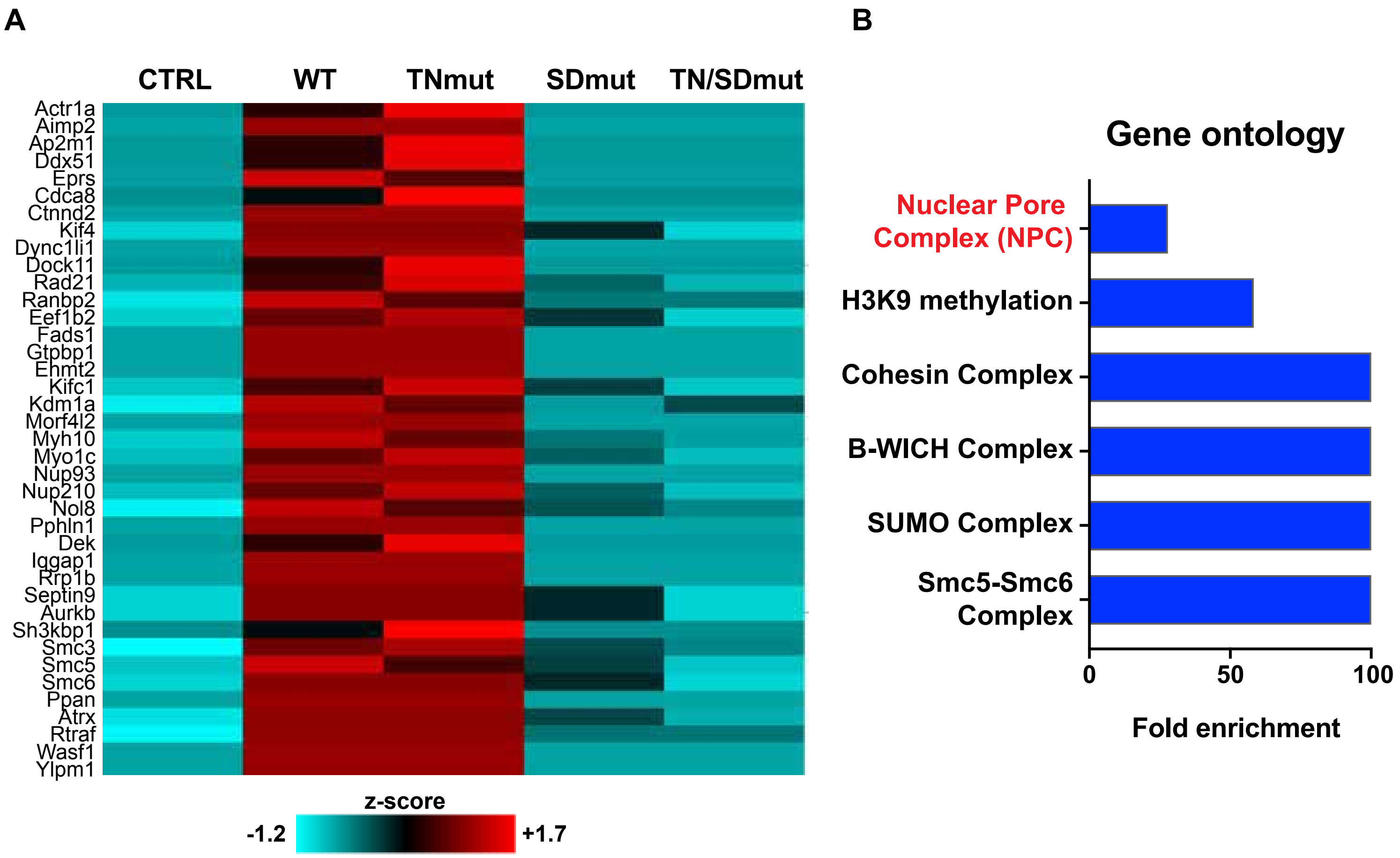

Figure S7. Mass spectrometry in MIN6 cells reveals interactions between NKX2.2, the Nuclear Pore Complex (NPC), and chromatin modifiers are reduced by mutation of the SD domain. (A) Heatmap of spectra counts for proteins that showed decreased interaction with wildtype NKX2.2 (WT) when the SD domain (SDmut) or both the SD and TN domain (SD/TNmut) were mutated, but not when the TN domain was mutated alone (TNmut). No interactions were detected in samples transfected with an empty control plasmid (CTRL). (B) Gene ontology analysis for proteins identified in A.
