# Supplementary material for: Major β cell-specific functions of NKX2.2 are mediated via the NK2-specific domain
Source: Genes Dev. 2023 Jun 1;37(11-12):490–504. doi: 10.1101/gad.350569.123 (PMC10393193; doi:10.1101/gad.350569.123)
Supplement: Supplemental Material [file supp_gad.350569.123_Supplemental_Fig_S8.pdf]

Abarinov Figure S8.

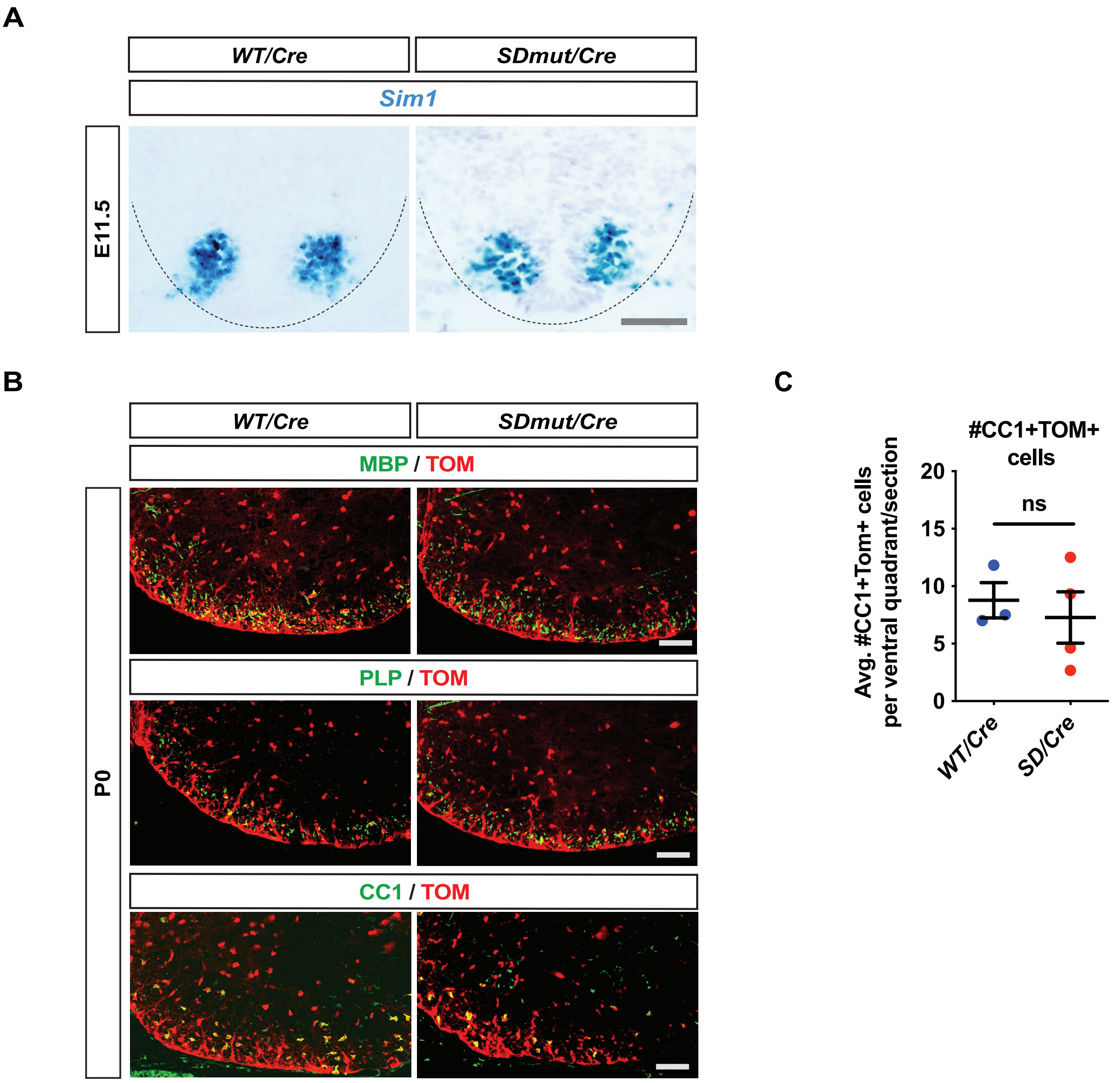

Figure S8. V3 interneurons and mature oligodendrocytes (OLGs) are generated normally in *Nkx2.2*$^{SDmut/Cre}$ animals at E11.5 and P0, respectively. (A) *Sim1 in situ* hybridization shows similar expression between *Nkx2.2*$^{Cre/+}$ (WT/Cre) and *Nkx2.2*$^{SDmut/Cre}$ (SDmut/Cre) E11.5 embryos. (B) Expression of mature OLG markers—MBP, PLP, and CC1—appears similar in *Nkx2.2*$^{Cre/+}$ *and Nkx2.2*$^{SDmut/Cre}$ P0 neonates. (C) Quantification of CC1+TOM+ cells from C reveals no significant differences between control and mutant mice. Data are presented as mean ± SEM. ns, not significant. n's are indicated by data points. Scale bars represent 50µm.
