# Supplementary material for: Major β cell-specific functions of NKX2.2 are mediated via the NK2-specific domain
Source: Genes Dev. 2023 Jun 1;37(11-12):490–504. doi: 10.1101/gad.350569.123 (PMC10393193; doi:10.1101/gad.350569.123)
Supplement: Supplemental Material [file supp_gad.350569.123_Supplemental_Fig_S9.pdf]

Abarinov Figure S9.

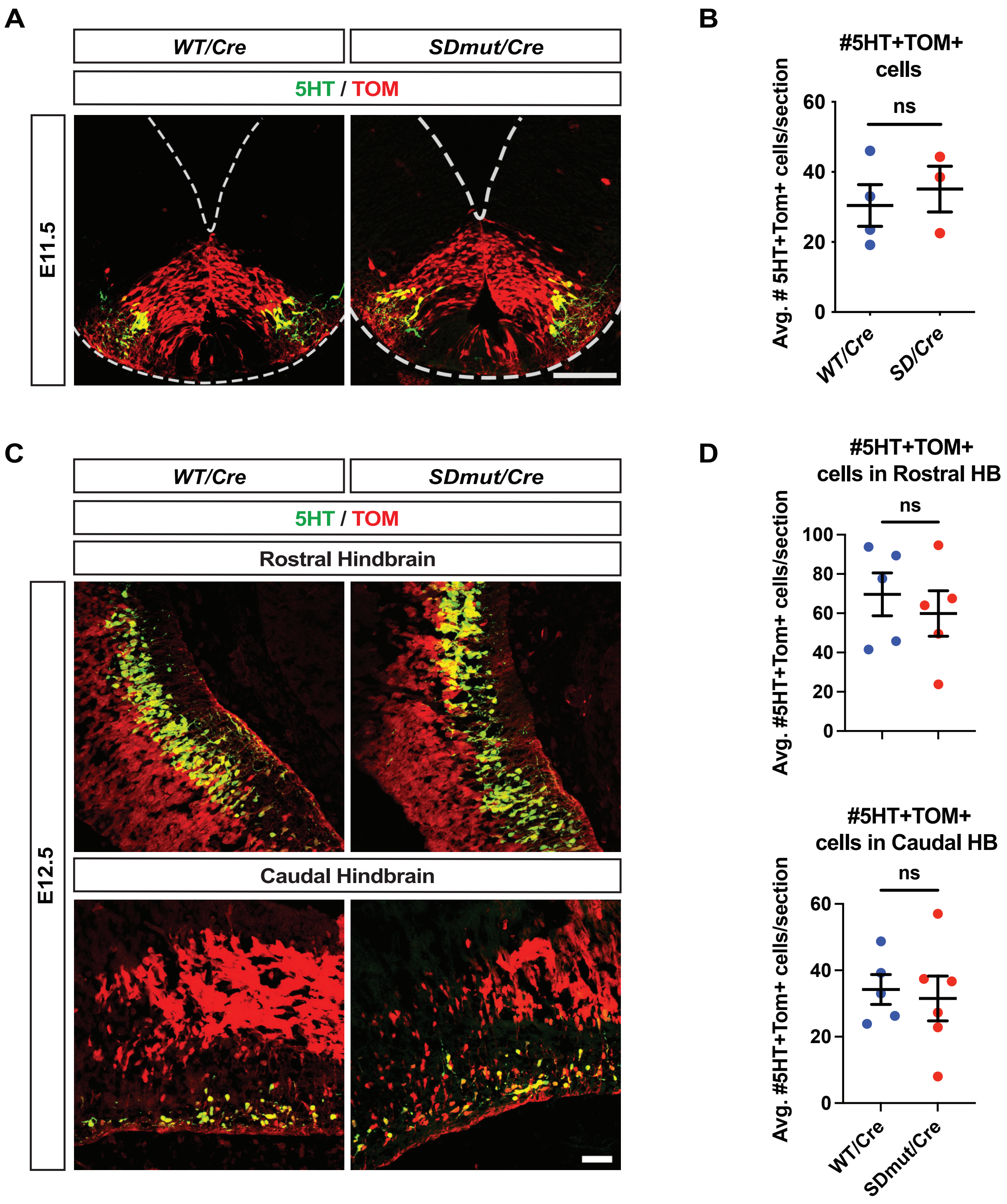

Figure S9. Serotonergic (5HT) neurons are generated normally in *Nkx2.2*$^{SDmut/Cre}$ embryos. (A-B) Immunostaining (A) and quantification (B) of 5HT+TOM+ cells in E11.5 hindbrains (HBs) of *Nkx2.2*$^{Cre/+}$ (WT/Cre) and *Nkx2.2*$^{SDmut/Cre}$ (SDmut/Cre) embryos. (C-D) Expression (C) and quantification (D) of 5HT+TOM+ cells in the rostral (top) and caudal (bottom) hindbrains of E12.5 *Nkx2.2*$^{Cre/+}$ and *Nkx2.2*$^{SDmut/Cre}$ animals. No significant differences in 5HT+TOM+ cell numbers were detected. Data are presented as mean ± SEM. ns, not significant. n's are indicated by data points. Scale bars represent 50μm.
