# Supplementary material for: Major β cell-specific functions of NKX2.2 are mediated via the NK2-specific domain
Source: Genes Dev. 2023 Jun 1;37(11-12):490–504. doi: 10.1101/gad.350569.123 (PMC10393193; doi:10.1101/gad.350569.123)
Supplement: Supplemental Material [file supp_gad.350569.123_Supplemental_Fig_S10_legend.pdf]

Figure S10. *In vitro* differentiation of iNKX2.2 and iSDmut mouse embryonic stem cells (ESCs) recapitulates *in vivo* development. (A) Top: Schematic of transgenic wildtype (iNKX2.2) and SD-mutant (iSDmut) *Nkx2.2* alleles. Both alleles are tagged by FLAG and are under the control of a DOX-responsive element (TRE). The *Nkx2.2*^{SDmut} allele is the same as that depicted in Fig. 1B. Bottom: Overview of ESC-directed motor neuron (MN) differentiation. Retinoic acid (RA) and Smoothen Agonist (SAG) are added at the primitive endoderm (PE) stage on Day 2 in order to initiate OLIG2 expression and motor neuron progenitor (pMN) specification beginning on Day 3. pMN cells differentiate into postmitotic HB9+ MNs by Day 6. To induce NKX2.2 expression and thereby disrupt MN differentiation, DOX is added to nascent OLIG2+ pMN cells late on Day 3. (B) NKX2.2 and OLIG2 expression at 2, 4, 8, and 16 hours after addition of DOX to iNKX2.2 cells differentiated toward the MN cell fate. iNKX2.2 cells to which DOX was not added served as the control (CTRL). (C) Schematic summarizing how the *in vitro* differentiation protocol (bottom) mimics *in vivo* development (top) as NKX2.2 is initially induced in early OLIG2-expressing pMN progenitors to disrupt MN differentiation in both conditions. (D) Expression of FLAG, OLIG2, and HB9 in DOX-treated iNKX2.2 and iSDmut embryoid bodies compared to control (CTRL) cells without DOX. OLIG2 and HB9 expression is abolished by induction of both wildtype and SD-mu-tant NKX2.2. (E) RNA-seq analysis described in Fig. 6 reveals induction of *Nkx2.2* expression is similar between iNKX2.2 and iSDmut cells on Day 4. Data are presented as mean ± SEM. ns, not significant. n = 3. Scale bar represents 50μm.
