# Supplementary material for: Major β cell-specific functions of NKX2.2 are mediated via the NK2-specific domain
Source: Genes Dev. 2023 Jun 1;37(11-12):490–504. doi: 10.1101/gad.350569.123 (PMC10393193; doi:10.1101/gad.350569.123)
Supplement: Supplemental Material [file supp_gad.350569.123_Supplemental_tables.pdf]

Supplementary Table 1. List of primary and secondary antibodies used in this study.

Pancreas				
**Antibody**	**Species**	**Source**	**Catalog#**	**Dilution**
Insulin	Rabbit	Cell Signaling Technology	3014	1:500
Insulin	Guinea Pig	Agilent / DAKO	IR00261-2	1:10
Glucagon	Rabbit	Cell Signaling Technologies	2760S	1:250
Glucagon	Goat	Santa Cruz	sc-7779	1:200
Somatostatin	Rat	Abcam	ab30788	1:500
Ghrelin	Goat	Santa Cruz	sc-0368	1:500
ChgA	Rabbit	Millipore	AB9254	1:500
Nkx2.2	Rabbit	Sigma Prestige	HPA003468	1:100
Nkx6.1	Goat	R&D	AF5857	1:1000
Nkx6.1	Rabbit	Beta Cell Biology Consortium	ab1069	1:800
MafA	Rabbit	Bethyl	IHC-00352	1:50 (P2) 1:500 (E18.5)
MafB	Rabbit	Bethyl	A700-046	1:100
Pdx1	Rabbit	Millipore	07-696	1:1000
Pdx1	Goat	Millipore	06-1385	1:500
GLUT2	Rabbit	Millipore	07-1402	1:500
**CNS**				
**Antibody**	**Species**	**Source**	**Catalog#**	**Dilution**
Nkx2.2	Mouse	DSHB	74.5A5	1:100
Olig2	Rabbit	Millipore	AB9610	1:1,000
Hb9	Guinea Pig	Generous gift from Thomas M. Jessell and Susan Morton (Columbia U.)	N/A	1:5,000
MBP	Mouse	Abcam	ab11159	1:500
PLP	Mouse	Abcam	ab9311	1:500
CC1	Mouse	Calbiochem	OP44-100UG	1:500
5HT	Rabbit	Sigma	S5545-25UL	1:500
Flag	Mouse	Sigma	F1804	1:100
**Secondary antibodies**				
**Antibody**	**Channel**	**Source**	**Catalog#**	**Dilution**
Donkey anti guinea pig	488	Jackson Immuno Research	706-545-148	1:500
Donkey anti	594	Invitrogen	A21207	1:500

rabbit				
Donkey anti goat	697	Invitrogen	A21447	1:500
Donkey anti mouse	488	Invitrogen	A21202	1:500
Donkey anti rat	594	Invitrogen	A21209	1:500

Supplementary Table 2. List of TaqMan probes used in this study.

**Gene**	**Assay ID**
Ins1	Mm01950294_s1
Ins2	Mm00731595_gH
Gcg	Mm00801714_m1
Ghrl	Mm00445450_m1
Sst	Mm00436671_m1
Gast	Mm00439059_g1
ChgA	Mm00514341_m1
Nkx6.1	Mm00454961_m1
Pdx1	Mm00435565_m1
Arx	Mm00545903_m1
Glut2	Mm00446229_m1
β-actin	Mm00607939_s1

Supplementary Table 3. NKX2.2 protein interactions reduced by mutation of the SD domain specifically. MYC-tagged wildtype (WT), TN domain-mutated (TNmut), SD domain-mutated (SDmut), and TN-mutated and SD-mutated (TNmut/SDmut) *Nkx2.2* constructs were expressed in MIN6 cells. A vector containing the MYC tag but no *Nkx2.2* sequence served as the control (CTRL). Mass spectrometry spectra counts for proteins that interacted with WT NKX2.2 and TNmut NKX2.2 but showed reduced interactions (lower spectra counts) with SDmut NKX2.2 and TNmut/SDmut NKX2.2 are presented. NKX2.2 counts for each condition are shown in red for reference.

Protein name	Accession #	Symbol	CTRL	WT	TNmut	SDmut	TNmut/SDmut
**Homeobox protein NKX2.2**	**P42586**	**Nkx2.2**	**0**	**31**	**22**	**25**	**21**
Alpha-centractin	P61164	Actr1a	0	1	2	0	0
Aminoacyl tRNA synthase complex-interacting multifunctional protein 2	Q8R010	Aimp2	0	1	1	0	0
AP-2 complex subunit mu	P84091	Ap2m1	0	1	2	0	0
ATP-dependent RNA helicase DDX51	Q6P9R1	Ddx51	0	1	2	0	0
Bifunctional aminoacyl-tRNA synthetase	Q8CGC7	Eprs	0	3	2	0	0
Borealin	Q8BHX3	Cdca8	0	1	3	0	0
Catenin delta-2 ctnnd2	O35927	Ctnnd2	0	1	1	0	0
Chromosome-associated kinesin KIF4	P33174	Kif4	0	2	2	1	0
Cytoplasmic dynein 1 light intermediate chain 1	Q8R1Q8	Dync1li1	0	1	1	0	0
Dedicator of cytokinesis protein 11	A2AF47	Dock11	0	1	2	0	0
Double-strand-break repair protein rad21 homolog	Q61550	Rad21	0	3	5	1	0
E3 SUMO-protein ligase RanBP2	Q9ERU9	Ranbp2	0	4	3	1	1
Elongation factor 1-beta	O70251	Eef1b2	0	4	5	2	0
Fatty acid desaturase 1	Q920L1	Fads1	0	1	1	0	0
GTP-binding protein 1	O08582	Gtpbp1	0	1	1	0	0
Histone-lysine N-methyltransferase, H3 lysine-9 specific 3 EHMT2	Q9Z148	Ehmt2	0	1	1	0	0
Kinesin-like protein KIFC1	Q9QWT9	Kifc1	0	2	3	1	0
Lysine-specific histone demethylase 1A	Q6ZQ88	Kdm1a	0	5	4	1	2
Mortality factor 4-like protein 2	Q9R0Q4	Morf4l2	0	1	1	0	0
Myosin-10	Q61879	Myh10	0	9	7	2	1
Myosin-Ic	Q9WTI7	Myo1c	0	3	4	1	0
Nuclear pore complex protein Nup93	Q8BJ71	Nup93	0	1	1	0	0
Nuclear pore membrane glycoprotein 210 Nup210	Q9QY81	Nup210	0	3	4	1	0
Nucleolar protein 8	Q3UHX0	Nol8	0	8	6	3	2
Periphilin-1	Q8K2H1	Pphln1	0	1	1	0	0
Protein DEK	Q7TNV0	Dek	0	1	2	0	0
Ras GTPase-activating-like protein IQGAP1	Q9JKF1	Iqgap1	0	1	1	0	0
Ribosomal RNA processing protein 1 homolog B	Q91YK2	Rrp1b	0	1	1	0	0
Septin-9	Q80UG5	Septin9	0	4	4	2	0
Serine/threonine-protein kinase 12	O70126	Aurkb	0	2	2	1	0
SH3 domain-containing kinase-binding protein 1	Q8R550	Sh3kbp1	0	1	3	0	0
Structural maintenance of chromosomes protein 3	Q9CW03	Smc3	0	6	7	3	2
Structural maintenance of chromosomes protein 5	Q8CG46	Smc5	0	3	2	1	0
Structural maintenance of chromosomes protein 6	Q924W5	Smc6	0	2	2	1	0
Suppressor of SWI4 1 homolog PPAN	Q91YU8	Ppan	0	1	1	0	0
Transcriptional regulator ATRX	Q61687	Atrx	0	7	7	3	1
UPF0568 protein C14orf166 homolog	Q9CQE8	Rtraf	0	3	3	1	1
Wiskott-Aldrich syndrome protein family member 1	Q8R5H6	Wasf1	0	2	2	0	0
YLP motif-containing protein 1	Q9R0I7	Ylpm1	0	1	1	0	0
